# Glutamine-Expanded Ataxin-7 Alters TFTC/STAGA Recruitment and Chromatin Structure Leading to Photoreceptor Dysfunction

**DOI:** 10.1371/journal.pbio.0040067

**Published:** 2006-02-28

**Authors:** Dominique Helmlinger, Sara Hardy, Gretta Abou-Sleymane, Adrien Eberlin, Aaron B Bowman, Anne Gansmüller, Serge Picaud, Huda Y Zoghbi, Yvon Trottier, Làszlò Tora, Didier Devys

**Affiliations:** **1**Department of Molecular Pathology, Institut de Génétique et de Biologie Moléculaire et Cellulaire, CNRS/INSERM/ULP, Illkirch, France; **2**Department of Transcription, Institut de Génétique et de Biologie Moléculaire et Cellulaire, CNRS/INSERM/ULP, Illkirch, France; **3**Chaire de Génétique Humaine, Collège de France, Paris, France; **4**Howard Hughes Medical Institute, Department of Molecular and Human Genetics, and Department of Pediatrics, Baylor College of Medicine, Houston, Texas, United States of America; **5**Imaging Technology Platform, Institut de Génétique et de Biologie Moléculaire et Cellulaire, CNRS/INSERM/ULP, Strasbourg, France; **6**Laboratoire de Physiopathologie Cellulaire et Moléculaire de la Rétine, INSERM U-592, UPMC, Paris, France; University of California San DiegoUnited States of America

## Abstract

Spinocerebellar ataxia type 7 (SCA7) is one of several inherited neurodegenerative disorders caused by a polyglutamine (polyQ) expansion, but it is the only one in which the retina is affected. Increasing evidence suggests that transcriptional alterations contribute to polyQ pathogenesis, although the mechanism is unclear. We previously demonstrated that the*SCA7* gene product, ataxin-7 (ATXN7), is a subunit of the GCN5 histone acetyltransferase–containing coactivator complexes TFTC/STAGA. We show here that TFTC/STAGA complexes purified from SCA7 mice have normal TRRAP, GCN5, TAF12, and SPT3 levels and that their histone or nucleosomal acetylation activities are unaffected. However, rod photoreceptors from SCA7 mouse models showed severe chromatin decondensation. In agreement, polyQ-expanded ataxin-7 induced histone H3 hyperacetylation, resulting from an increased recruitment of TFTC/STAGA to specific promoters. Surprisingly, hyperacetylated genes were transcriptionally down-regulated, and expression analysis revealed that nearly all rod-specific genes were affected, leading to visual impairment in SCA7 mice. In conclusion, we describe here a set of events accounting for SCA7 pathogenesis in the retina, in which polyQ-expanded ATXN7 deregulated TFTC/STAGA recruitment to a subset of genes specifically expressed in rod photoreceptors, leading to chromatin alterations and consequent progressive loss of rod photoreceptor function.

## Abstract 2

A mouse model of Spinocerebellar ataxia type 7 (SCA7) offers new insight into transcriptional alterations that contribute to this neurodegenerative disorder.

## Introduction

Spinocerebellar ataxia type 7 (SCA7) is an autosomal dominant neurodegenerative disorder caused by expansion of an unstable CAG trinucleotide repeat encoding a polyglutamine (polyQ) stretch [1,2]. The expansion of a translated CAG repeat causes eight other progressive neurodegenerative diseases, including Huntington disease [3]. Expanded polyQ confers a toxic gain of function to otherwise unrelated proteins. Aggregation of expanded polyQ-containing proteins in the nucleus is a hallmark of these diseases. Despite these common properties, polyQ diseases also show distinctive features, which may reflect the unique protein context of the polyQ expansion in each disease. SCA7 can be distinguished from other polyQ diseases, as it is the only one affecting the retina leading to visual impairment and eventually to blindness [4].

We recently demonstrated that ataxin-7 (ATXN7), the protein mutated in SCA7, is a bona fide subunit of TFTC, theTATA-binding protein (TBP)-free TBP-associated factor (TAF)-containing complex, and STAGA, the SPT3/TAF9/GCN5 acetyltransferase complex [5]. These transcriptional co-activator complexes were shown to preferentially acetylate histone H3 in both free and nucleosomal contexts and to activate transcription on chromatin templates [6–8]. TFTC/STAGA complexes contain the GCN5 histone acetyltransferase (HAT), TRRAP, SPT, ADA proteins, and a subset of TAFs, but clearly lack TBP [9]. In contrast, the general transcription factor TFIID contains TBP and 14 TAFs, some of which are shared with TFTC/STAGA. TFTC/STAGA complexes are the mammalian homologs of the yeast SAGA (Spt/Ada/Gcn5) complex [10,11]. Genome-wide expression studies indicate that transcriptional regulation of approximately 10% of the yeast genome is predominantly regulated by SAGA and 90% by TFIID. Most SAGA-dominated genes were found stress inducible and highly regulated although TFIID controlled the expression of housekeeping genes [12]. In comparison with yeast SAGA, the mechanisms by which mammalian TFTC-like complexes regulate RNA polymerase II (Pol II) transcription remain elusive.

Early and severe transcriptional alterations were detected in various polyQ models [13]. Expanded polyQ-containing proteins have been proposed to interfere with the normal function of several transcription factors, notably by sequestering them to nuclear inclusions (NIs), but the exact mechanisms leading to transcriptional deregulation remain largely unknown. The mouse retina is a suitable neuronal tissue to perform transcriptional studies as rods represent around 97% of photoreceptors and more than 70% of all retinal cells [14]. In wild-type (WT) mice, the nuclei of rod photoreceptors display a very peculiar organization different from that of any other retinal neurons, with a thin rim of euchromatin surrounding a large heterochromatin territory [15]. Chromatin plays a pivotal role in regulating transcription, suggesting that rod nuclear organization specifies its gene expression profile. In particular, genes encoding components of the phototransduction cascade are expressed specifically in rods, at remarkably high levels due to constant renewal of rod outer segments.

We previously generated transgenic mice specifically expressing normal (10Q) or mutant (90Q) ATXN7 in rod photoreceptors (hereafter called R7N and R7E lines, respectively) [16]. Mutant ATXN7 induced a progressive loss of segments and dramatic reduction of rod electrophysiological activity in R7E mice. Segment loss paralleled a progressive and severe down-regulation of*Rho* (rhodopsin), which was the earliest molecular anomaly detectable in these mice [17]. Similar observations were made in a SCA7 knock-in mouse model, which expresses mutant ATXN7 at endogenous levels [18], suggesting that loss of photoreceptor-specific gene expression is relevant to SCA7 retinopathy. Finally, recent studies performed in yeast or in transfected cells suggest that polyQ-expanded ATXN7 incorporation in TFTC/STAGA alters their composition and inhibits their nucleosomal HAT activity [19,20].

Here, we investigated the composition and activity of TFTC/STAGA complexes in vivo. We discovered that, in the retina of SCA7 mice, mutant TFTC/STAGA HAT complexes induced histone H3 hyperacetylation through their aberrant recruitment to promoters of rod photoreceptor-specific genes. Furthermore, rod nuclei showed chromatin decondensation which, surprisingly, correlated with severe transcriptional repression of the corresponding genes. Alteration of rod highly organized nuclear architecture disrupted the rod-specific expression profile through down-regulation of most genes whose expression is normally high and restricted to rods. Finally, loss of expression of most genes required for phototransduction provides a molecular explanation for the retinal degeneration observed in SCA7 mice.

## Results

### Severe Alteration of Rod Photoreceptor Nuclear Architecture in SCA7 Mice

R7E mice, which express mutant (90Q) ATXN7 under control of the rhodopsin promoter, have a normal lifespan due to restricted transgene expression in rod photoreceptors. This allowed us to show that mutant ATXN7-induced retinopathy is characterized by long-term and complete dysfunction of rods [17]. We have now investigated more thoroughly the ultrastructural changes accompanying prolonged rod dysfunction by electron microscopy of WT, R7N, and R7E retinas at different ages. Transgenic lines R7N.C and R7E.A were used throughout this study [16].

**Figure 1:**
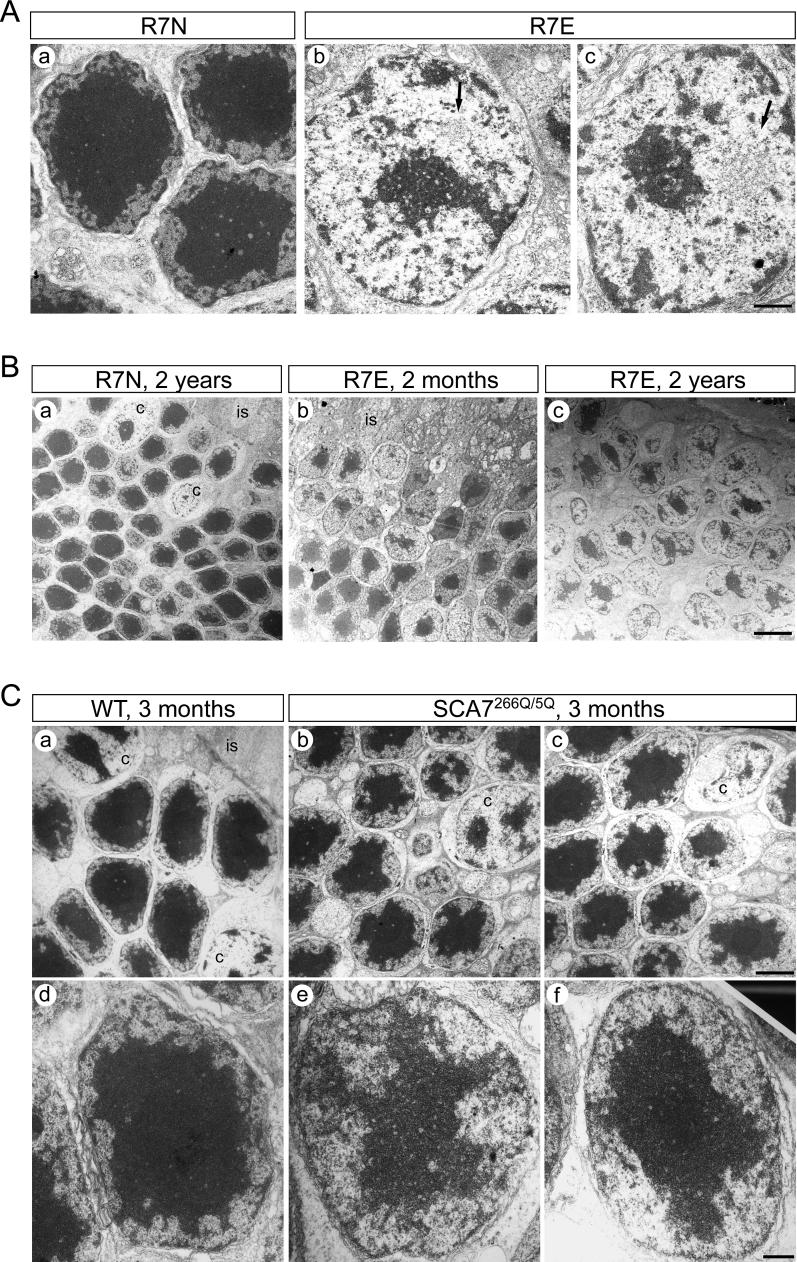
Severe Reorganization of Chromatin Territories in Rod Photoreceptors from SCA7 Mice (A) Ultrastructure of rod nuclei in R7E mice. Electron microscopy micrographs from 2-y-old R7N (panel a) and two distinct R7E (panels b and c) animals are shown. In R7N mice, rod nuclei are characterized by a large territory of centrally located heterochromatin. In R7E mice, rod nuclei were bigger and rounder, and displayed much more euchromatin, which appears lightly stained by electrons. Arrows indicate pale grey structures likely resulting from aggregation of mutant ATXN7 in rods from R7E mice. Scale bar represents 1 μm. (B) Ultrastructure of photoreceptor nuclei during disease progression in R7E mice. Electron microscopy micrographs from 2-y-old R7N (panel a), 2-mo-old R7E (panel b), and 2-y-old R7E (panel c) animals are shown. This time-course analysis revealed that alterations of rod nuclear architecture progressively worsened as retinopathy progressed in R7E mice. Cone photoreceptor nuclei (c), which are found in the outermost part of the ONL adjacent to inner segments (is), present a different architecture with several heterochromatin clumps surrounded by more euchromatin. As evidenced in panel c, rod nuclei in R7E mice look highly similar to cone nuclei. Scale bar represents 5 μm. (C) Ultrastructure of photoreceptor nuclei in SCA7 knock-in mice. Electron microsocopy micrographs from 3-mo-old WT (panels a and d) and two distinct age-matched SCA^7266Q/5Q^ (panels b, c, e, and f) animals are shown. Morphological appearance of the central densely stained heterochromatin territory is altered in mutant mice. At higher magnification (lower panels [e–f]), rod nuclei from heterozygous knock-in animals are slightly larger and contain more euchromatin than nuclei from control mice. Cone nuclei (c) and photoreceptor inner segments (is) are depicted. Scale bars represent 2 μm in upper panels (a–c) and 0.5 μm in lower panels (e–f).

The most surprising abnormality was a striking modification of rod nuclear architecture (Figure 1). In WT mice, rods have small nuclei (4–5 μm diameter) with a characteristic polyhedral shape and contain a single, large clump of heterochromatin surrounded by a sparse rim of euchromatin (Figure 1C, panels a and d). R7N rod photoreceptors were indistinguishable from those of WT mice (Figure 1A, panel a), consistent with the absence of phenotype in this line [16,17]. In marked contrast, rods from R7E mice showed a dramatic chromatin decondensation, associated with a massive increase in nuclear volume (Figure 1A, panels b and c). Consequently, rod nuclei from R7E mice showed much more euchromatin and resembled cone nuclei or that of neurons from the inner nuclear layer. However, these abnormal photoreceptors were undoubtedly identified as rods because a fibrillar structure was observed in the decondensed chromatin territory (arrows inFigure 1A, panels b and c), which likely corresponded to aggregated mutant ATXN7. Immunofluorescence staining confirmed the presence of a single NI in weakly DAPI-stained territories in rods from aged R7E mice (Figure S1A). This aberrant chromatin alteration was a progressive phenomenon as it was already observable in 2-mo-old R7E mice, although less prominent and heterogeneous (Figure 1B, panel b). At 2 y of age, almost all nuclei were larger and rounder, and the spacing between nuclei was greater than in WT retina (Figure 1B, panel c). Finally, we noticed a normal retinal epithelium appearance despite dramatic loss of segments (Figure S2), indicating that rods from R7E mice are defective in segment renewal.

### Comparable Chromatin Remodeling in Rods from SCA7 Knock-In Mice

We then tested whether this nuclear phenotype was relevant to SCA7 retinopathy or due to overexpression of mutant ATXN7 in R7E mice. We performed electron microscopy of retina from 3-mo-old SCA7 heterozygous knock-in mice (Sca^7266Q/5Q^) which present a retinopathy comparable to that occurring in R7E animals [18]. Rod nuclear architecture was altered in Sca^7266Q/5Q^ mice when compared to WT littermates (Figure 1C). Rod nuclei from Sca^7266Q/5Q^ mice contained more euchromatin and displayed a reduced and disorganized heterochromatin territory (compare panels b, c, e, and f with panels a and d inFigure 1C). We could observe a small, pale fibrillar structure in the decondensed chromatin territory (Figure S3), which likely resulted from aggregation of mutant ATXN7 into NIs. Although comparable, chromatin decondensation appeared less severe in heterozygous Sca^7266Q/5Q^ animals than in age-matched R7E mice. Premature death of Sca^7266Q/5Q^ mice precluded analysis of the extent of chromatin remodeling at later stages, when the nuclear phenotype was prominent in R7E mice. These data together show that significant chromatin alterations occurred in rod nuclei of both SCA7 transgenic and knock-in mouse models, further suggesting that this aberrant nuclear phenotype is relevant to polyQ-expanded ATXN7-induced retinopathy.

### Aberrant Rod Chromatin Remodeling Is Correlated with Rod Dysfunction

**Figure 2:**
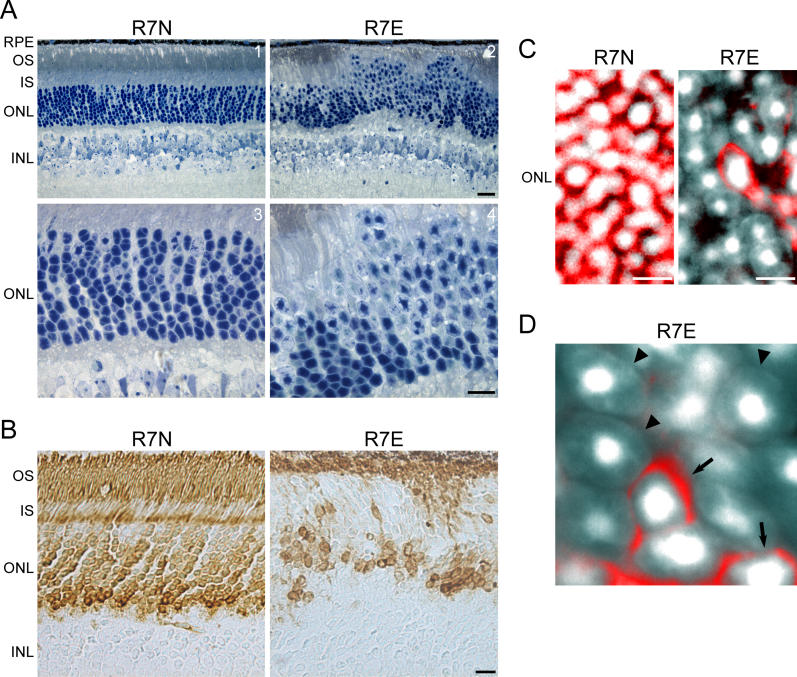
Chromatin Remodeling in Rod Nuclei Paralleled Segment Loss and*Rho* Down-Regulation in SCA7 Mice (A) Histological examination of retina from 2-mo-old R7N (panels 1 and 3) and R7E (panels 2 and 4) mice. Toluidin-blue staining of semi-thin sections revealed a discontinuous pattern of segment loss which paralleled an irregular appearance of rod chromatin decondensation in R7E animals (upper panels). High magnification micrographs (lower panels) showed that rods with preserved nuclear morphology are connected with inner segments of normal appearance. Scale bars represent 20 μm in upper panels and 10 μm in lower panels. (B–D) Rhodopsin immunolocalization in 2-mo-old R7N and R7E retina. (B) Immunohistochemistry of paraffin-embedded sections was performed using an anti-rhodopsin antibody (4D2). In R7E retina, residual staining was observed in the innermost part of the ONL and in folds in which normal condensed rod nuclei were typically located. Scale bar represents 10 μm. (C and D) Immunofluorescence of retinal cryosections were performed using an anti-rhodopsin antibody (4D2) and DAPI to visualize rod nuclei. Images were collected by confocal imaging analysis, and merged images (rhodopsin in red, DAPI in grey scale) are shown. (C) Very few cells retained rhodopsin staining, which circumscribes the nucleus in the ONL. (D) Higher magnification images demonstrated that residual staining was exclusively found in rods showing a preserved nuclear morphology (arrows), whereas no rhodopsin could be detected in rods showing an altered chromatin organization (arrowheads). Scale bar represents 5 μm. INL, inner nuclear layer; IS, inner segments; RPE, retinal pigment epithelium.

In early stages of the R7E retinal phenotype (at 2 mo), different degrees of chromatin decondensation were observed by electron microscopy (Figure 1B) and by histological examination of toluidin-blue stained sections (Figure 2A). Strikingly, normal condensed rod nuclei were always connected with preserved inner and outer segments, whereas areas with abnormal decondensed rod nuclei were associated with segment thinning (Figure 2A, bottom right panel). Interestingly, areas with remaining segments were occasionally found associated with slightly decondensed nuclei, wheres areas without any segments left were never associated with normal condensed nuclei. Furthermore, mild alterations of chromatin condensation could be detected on toluidin blue–stained sections of 4-wk-old animals (Figure S1B), before the onset of R7E retinopathy. These two observations suggested that the aberrant chromatin decondensation might be a primary event preceding the loss of segments and rod dysfunction.

**Figure 3:**
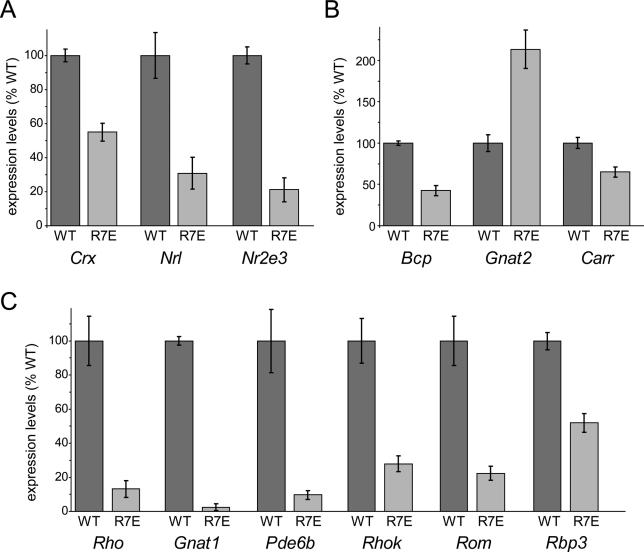
Comprehensive Characterization of Specific Transcriptional Alterations in the Retina from SCA7 Mice mRNA levels were quantified by real-time PCR of reverse transcribed RNA from 2-mo-old WT and R7E retina. Expression levels are represented as a percentage of the mean of WT littermate mice after normalization to*Ppia* or*Arbp* levels. In each panel, each bar represents the mean value ± the standard error of the mean (SEM) (*n* = 3–6). (A) Down-regulation of genes encoding transcription factors,*Crx, Nrl,* and*Nr2e3,* involved in photoreceptor differentiation and maintenance. (B) Expression analysis of cone-specific genes,*Bcp, Gnat2,* and*Carr,* in WT and R7E animals. (C) Severe down-regulation of rod-specific genes,*Rho, Gnat1, Pde6b, Rhok, Rom,* and*Rbp3,* encoding components of the phototransduction machinery.

Immunohistology using an anti-rhodopsin antibody revealed a striking, patchy down-regulation of*Rho* expression in 2-mo-old R7E mice (Figure 2B). Immunofluorescence showed that nuclei of rhodopsin-negative rods always displayed severe chromatin decondensation (Figure 2C and arrowheads inFigure 2D), whereas nuclei from immunopositive rods had a preserved chromatin architecture (Figure 2C and arrows inFigure 2D). Thus, rods undergoing chromatin decondensation have completely silenced rhodopsin expression, and the 10% residual*Rho* mRNA levels detected by RT-PCR (Figure 3) at 2 mo of age are likely due to a small proportion of rods with a preserved chromatin structure. Altogether these results demonstrate that polyQ-expanded ATXN7 induces a dramatic chromatin alteration that correlates with extinction of*Rho* expression.

### Reduced mRNA Levels of Rod-Specific Genes in SCA7 Mice

Activation of transcription is usually associated with chromatin decondensation. However, we found that*Rho* down-regulation correlated with chromatin decondensation. We thus tested to what extent such an important change in chromatin organization would affect the gene expression profile of rod photoreceptors. Because the rod nuclear architecture in aged R7E mice was similar to that normally seen in cone photoreceptors (Figure 1B), we analyzed expression levels of transcription factors specifically expressed and involved in rod versus cone fate specification.*Crx,* a homeobox transcription activator,*Nrl,* a basic motif-leucine zipper transcription activator, and*Nr2e3,* an orphan nuclear receptor, are essential for rod and cone differentiation by controlling photoreceptor gene expression [21–26]. Quantitative RT-PCR revealed decreased mRNA levels of*Crx* (2-fold, 55% residual mRNA levels),*Nrl* (3-fold, 31%), and*Nr2e3* (5-fold, 21%) in 2-mo-old R7E retina (Figure 3A), suggesting an alteration of photoreceptor differentiation in adult R7E mice. We then quantified mRNA levels of genes encoding cone- or rod-specific markers, but we did not observe a consistent up-regulation of genes encoding components of cone phototransduction cascade. In 2-mo-old R7E mice, blue cone opsin*(Bcp)* and cone arrestin*(Carr)* were down-regulated (43% and 65% residual levels, respectively), whereas cone transducin*(Gnat2)* was up-regulated by 113% (Figure 3B).

Importantly however, we found a severe down-regulation of most rod-specific genes tested (12 out of 13). In 2-mo-old R7E mice, quantitative RT-PCR showed a down-regulation of rhodopsin (*Rho,* 13% residual mRNA levels), rod transducin (*Gnat1,* 3%), rod phosphodiesterase β-subunit (*Pde6b,* 10%), rhodopsin kinase (*Rhok,* 28%), Rom-1 (*Rom,* 23%), and Irbp (*Rbp3,* 52%) (Figure 3C andTable S1). Moreover, using Northern blot experiments, we observed comparable reduction of other rod-specific mRNA levels (Table S1), namely phosducin*(Pdc)*, rod cGMP-gated channel*(Cncg),* rod arrestin*(Sag),* guanylate cyclase activator*(Guca),* recoverin*(Rcvrn),* and peripherin 2*(Prph2).* Five distinct housekeeping genes*(Ppia, Gapd, Arbp, Tuba1,* and*Hprt)* showed no differential expression between R7E and WT mice (see figure legends; data not shown).

Finally, we extended our analysis of transcriptional deregulation to the level of the whole transcriptome. Hybridization of MOE 430A Affymetrix microarrays that contained approximately 22,000 mouse transcripts using total RNA extracted from WT, R7N, and R7E mice at 2 mo of age, revealed that polyQ-expanded ATXN7 does not induce a global alteration of mRNA levels, as less than 1% of genes were deregulated with a 2-fold threshold (*p* ≤ 0.015, Mann-Whitney test) [27]. Importantly, when down-regulated genes were classified according to their expression levels in control retina, 29 of the 50 most highly expressed genes were found to be only expressed in the retina. Thus, polyQ-expanded ATXN7 preferentially down-regulated genes with a retina-restricted expression pattern, accounting for the visual impairment observed in SCA7 mice [17,18]. Finally, we did not find up-regulation of genes encoding factors regulating chromatin structure or accessibility. This aberrant expression profile suggests that rods from R7E mice have completely lost their differentiated state, but did not trans-differentiate into mature cone photoreceptors.

### Down-Regulated Rod-Specific Genes Are Transcriptionally Altered in SCA7 Mice

Because chromatin decondensation has been typically associated with active transcription, we tested whether the decreased mRNA levels of rod-specific genes were indeed due to a reduced transcriptional activity of these genes. We performed chromatin immunoprecipitation (ChIP) experiments using formaldehyde cross-linked chromatin extracts prepared from 2-mo-old control (WT or R7N) and R7E retina. We used an antibody raised against the C-terminal domain of the largest subunit of RNA Pol II in ChIP experiments to quantify the relative RNA Pol II occupancy at promoters and coding regions of down-regulated genes. Immunoprecipitated or input DNA amounts were quantified by real-time PCR using primers covering promoter regions from five rod-specific genes*(Rho, Pde6b, Rbp3, Crx,* and*Nrl)* and from a ubiquitously expressed gene,*Ncl* (nucleolin).

**Figure 4:**
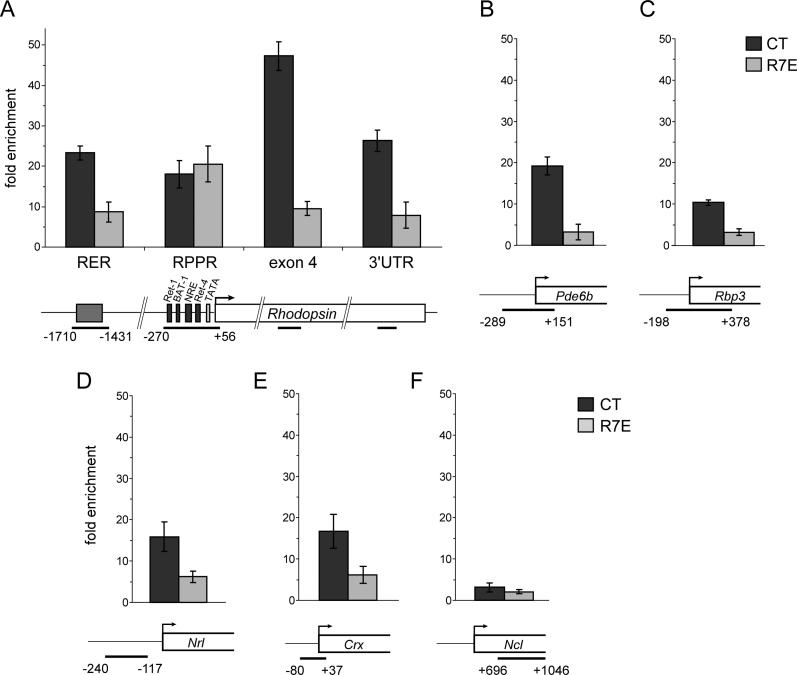
Decreased RNA Polymerase II Occupancy at Promoters and Coding Regions of Genes Down-Regulated in SCA7 Mice (A–F) ChIP assays were performed using formaldehyde-fixed chromatin extracts of retina from 2-mo-old control (WT or R7N; dark grey) and R7E (light grey) mice. Specific regions within the different genes were analyzed, as depicted; within the*Rho* gene, each amplified regions were separated by 1.5 kb at least. Each bar represents the mean value ± SEM (*n* = 4–6). RNA polymerase II occupancy, and thus transcriptional activity, was assessed using an antibody directed against the heptapeptide repeat of its C-terminal domain (Pol II CTD). All genes down-regulated at the mRNA level in R7E retina*(Rho, Pde6b, Rbp3, Nrl,* and*Crx)* showed marked decrease in Pol II occupancy at their promoters.

All down-regulated genes analyzed showed reduction of RNA Pol II occupancy at the corresponding promoter regions (Figure 4A–E). Furthermore, RNA Pol II recruitment at the*Rho* enhancer region [28], at the coding sequence (exon 4) and at the 3′UTR of*Rho,* was strongly reduced in R7E mice (Figure 4A). No differences were detected in the coding region of a house-keeping gene*(Ncl),* encoding nucleolin (Figure 4F). Thus, the observed reduction of rod-specific mRNA levels resulted from decreased transcriptional activity of these genes.

### Specific Compartmentalization of Expressed Genes in Rod Photoreceptor Nuclei

Surprisingly, the severe chromatin decondensation in R7E retina did not induce a global alteration of the rod transcriptome, but rather a specific defect in expression of a small subset of genes. We hypothesized that these genes, normally highly expressed, would localize to peripheral euchromatin in normal rods and that this specific compartmentalization would be disrupted by the chromatin alterations that occur in rods from SCA7 mice. Accordingly, immunostaining of WT retina using antibodies against transcription factors (ATXN7, RNA Pol II, TBP, TAF10, Crx, and CBP) and chromatin-associated proteins (HP1γ, HMGI(Y), Rb1, and acetyl-histone H3) revealed that these proteins were always excluded from the heterochromatic region, showing a characteristic ring-like appearance (Figure S4, and see [29]).

**Figure 5:**
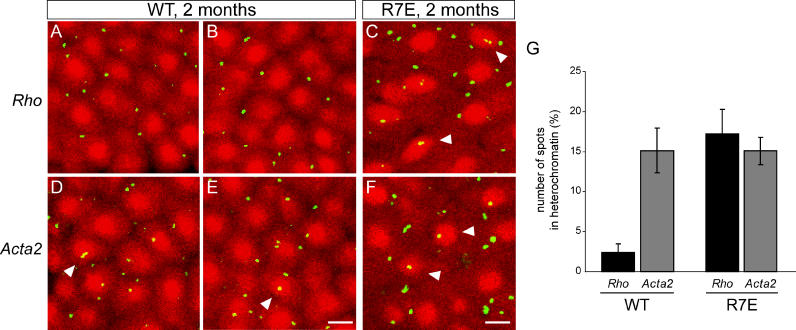
Loss of a Specific Gene Compartmentalization in Rods from SCA7 Mice (A–F) BAC probes containing*Rho* (A–C) or*Acta2* (D–F) genes were hybridized to retinal cryosections from 2-mo-old WT (A), (B), (D), and (E) or R7E (C) and (F) animals. FISH signals appear in green whereas DAPI-stained photoreceptor nuclei were pseudo-colored in red for better visualization. Merged images were collected by confocal imaging analysis and showed co-localization of*Acta2* within the densely DAPI-stained heterochromatin region in WT rod nuclei (arrowheads in [D] and [E]). By contrast,*Rho* was excluded from this compact heterochromatin region. In R7E rod nuclei,*Rho* and*Acta2* showed a comparable random pattern of intranuclear distribution. Both could be found co-localizing within the central densely DAPI-stained region (arrowheads in [C] and [F]). Scale bars represent 2 μm. (G) Distribution of*Rho* and*Acta2* between heterochromatin and euchromatin territories in WT and R7E rod nuclei was estimated by counting the number of spots detected in the densely DAPI-stained region. Counting was performed on the projection of four consecutive*z* stacks (1.2 μm between each stack) taken through the retinal section such that only entire rod nuclei were included in the counting. More than 500 nuclei were analyzed, and each bar represents the mean value ± SEM of three independent experiments performed on two different animals.

We used interphase fluorescent in situ hybridization (FISH) of mouse retina sections to investigate the nuclear localization of the gene encoding rhodopsin*(Rho)* versus a gene not expressed in rods, the vascular smooth muscle alpha-actin gene*(Acta2)*. BAC probe specificity was confirmed by hybridization of metaphase chromosome spreads from mouse embryonic stem cells (Figure S5).*Rho* and*Acta2* probes were hybridized to retinal sections from 2-mo-old WT and R7E mice, and each probe revealed one or two specific spots in virtually all nuclei (Figure 5). Confocal analysis revealed that the*Rho* gene was almost never found inside the central, densely DAPI-stained chromatin territory in WT rod nuclei (Figure 5A and5B). In contrast, the*Acta2* gene could be reproducibly detected within this region (arrowheads inFigure 5D and5E). Quantitative estimates of heterochromatin localization clearly differentiated the location of the*Acta2* and*Rho* genes, showing 15.1 ± 2.8% and 2.4 ± 1.1% of signals centrally located, respectively (Figure 5G). This difference is likely underestimated as the section treatment slightly modified the rod nuclear structure (seeMaterials and Methods).

This highly specific distribution was strikingly altered in rod nuclei from 2-mo-old R7E mice (Figure 5C and5F). Indeed, a similar proportion of FISH signals corresponding to*Rho* and*Acta2* genes could be detected in the densely DAPI-stained area (Figure 5G), indicating a. random, non-specific distribution of both expressed and non-expressed genes. We do not know at this point whether the proportion of rods showing a central FISH signal (around 16%) reflects the actual situation in vivo or is due to the intrinsic limit of the FISH technique. Altogether, these results suggested that the rod nuclear organization normally compartmentalizes rod-specific genes in peripheral euchromatin. However, in R7E mice, alteration of this compartmentalization presumably contributes to loss of the rod-specific gene expression profile.

### PolyQ-Expanded ATXN7 Incorporation in TFTC/STAGA Complexes Does Not Modify Their HAT Activity

**Figure 6:**
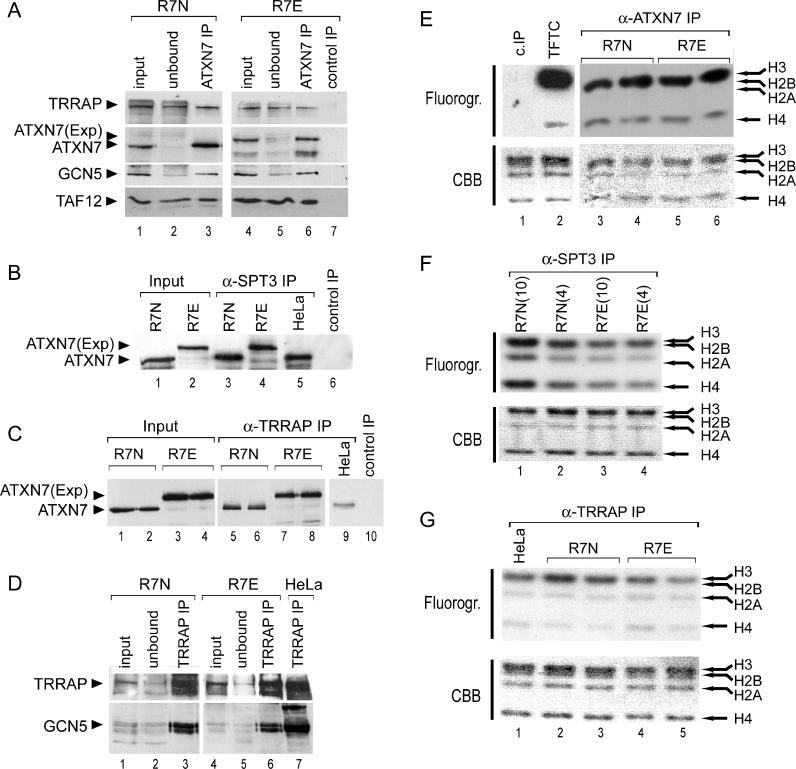
TFTC Subunit Composition and HAT Activity Are Not Altered by PolyQ-Expanded ATXN7 in R7E Retina (A) Western blot analysis of TFTC-type complexes immunopurified from 4-wk-old R7N or R7E retinal homogenates using an anti-ATXN7 antibody. ATXN7 IPs revealed comparable levels of Trrap, Gcn5, and Taf12 in complexes purified from R7N or R7E retina. (B and C) Whole retinal extracts from 4-wk-old R7N and R7E animals were immunoprecipitated with an anti-SPT3 (B) and anti-TRRAP antibody (C). Using the same antibodies, complexes were also immunoprecipitated from HeLa cell nuclear extract as positive controls. The retinal extracts (Input) and the immunopurified complexes were analyzed by immunoblotting with an anti-ATXN7 antibody. Complexes contained similar amounts of normal or mutant ATXN7. (D) Western blot analysis using anti-TRRAP and anti-GCN5 antibodies on complexes immunoprecipitated with an anti-TRRAP antibody from 10-wk-old R7N and R7E retinal homogenates. Using the same antibody, complexes were also immunoprecipitated from HeLa cell nuclear extract as positive controls. Comparable levels of Gcn5 and Trrap were detected in purified complexes from R7N and R7E retina. (E–G) HAT activities of immunopurified complexes bound to either the anti-ATXN7 pAb (E), anti-SPT3 mAb (F), or anti-TRRAP mAb beads (G) from R7N and R7E retina were measured by an in vitro HAT assay performed on free core histones. Histones were separated by SDS-PAGE and stained by Coomassie Brilliant Blue (CBB) and acetylated histones were visualized by fluorography (Fluorogr). The position of each histone is indicated. The pattern of histone acetylation was identical to that of a highly purified TFTC fraction used as a positive control. No histone acetylation could be detected when the antibody was omitted. The age of the mice are indicated in weeks in brackets. These results are representative of three independent experiments. Quantification of all HAT assays performed on R7N and R7E retina is provided inFigure S6A.

The discrepancy between chromatin and transcriptional abnormalities in R7E mice prompted us to analyze the effect of the polyQ expansion in ATXN7 on the TFTC HAT complex composition and activity. We previously reported that both normal and mutant ATXN7 can incorporate into TFTC using transfected HEK293T [5]. We have now extended this result by analyzing TFTC-type complexes purified from the retina of R7N and R7E transgenic animals, where the pathology normally appears in SCA7 patients. Co-immunoprecipitation experiments were performed using whole retinal extracts from 4-wk-old mice, an age at which mutant ATXN7 is soluble and not proteolytically processed [16]. First an anti-ATXN7 immunoprecipitation (IP) using R7N or R7E mice retinal extracts, retrieved bona fide TFTC subunits, such as Gcn5, Trrap, Taf10, and Taf12, together with normal or polyQ-expanded ATXN7 (Figure 6A, and data not shown). Reciprocally, when anti-TRRAP or anti-SPT3 antibodies were used, normal and mutant ATXN7 were equally incorporated into the Trrap- and Spt3-containing complexes (Figure 6B, lanes 3 and 4, andFigure 6C, lanes 5–9). We also tested whether the presence of mutant ATXN7 changed the incorporation of the histone acetylase subunit Gcn5 or of other subunits known to regulate TFTC HAT activity, such as Taf12 [30]. TFTC complexes prepared by an anti-ATXN7 or an anti-TRRAP IP from R7N and R7E mice contained about equal levels of Gcn5 and Taf12, as shown by Western blot analysis of immunopurified fractions (Figure 6A, lanes 3 and 6, andFigure 6D, lanes 3 and 6). Importantly, Gcn5 incorporation was not altered even in 10-wk-old mice (Figure 6D), an age at which severe chromatin changes were observed.

Next we tested whether the above-purified HAT complexes, containing either normal or mutant ATXN7 (Figure 6A–D), differentially acetylate free or nucleosomal histones in an in vitro acetylation test. TFTC-type complexes purified by an anti-AXN7 IP from the retina of R7N mice have the expected HAT activity, since they acetylate mainly histone H3 and, weakly, H4 as does the highly purified TFTC (Figure 6E, and see [6]). Histone acetylation pattern of complexes immunopurified from R7N or R7E retina were indistinguishable (Figure 6E, lanes 3–6). When Spt3- or Trrap-containing complexes were tested for their acetylation activity we did not observe a significant difference between complexes purified from retina of R7N or R7E mice at either 4 or 10 wk of age (Figure 6F, lanes 1–4, andFigure 6G, lanes 2–5). Quantification of the HAT activities of ATXN7- and Trrap-containing complexes revealed an equal histone acetylation activity of TFTC-type complexes purified from R7N and R7E retina (Figure S6A). Using an anti-SPT3 antibody, we observed a slight decrease (0.25-fold) of TFTC HAT activity in R7E mice (Figure S6A). We then carried out these assays using nucleosomal histones and consistently observed no significant differences between the HAT activities of complexes prepared from either R7N or R7E mice (Figure S6B andS6C). These data together suggest that polyQ-expanded ATXN7 does not significantly alter the in vitro HAT activity of TFTC-type complexes.

### Aberrant TFTC Recruitment to Promoters of Rod-Specific Genes in SCA7 Mice

We then tested whether the abnormal chromatin remodeling could result from a deregulation of TFTC recruitment to specific genes in rods from SCA7 mice. We performed ChIP experiments using chromatin extracts prepared from 2-mo-old control (WT or R7N) and R7E retina to quantify TFTC recruitment at genes down-regulated*(Rho, Pde6b, Rbp3, Crx,* and*Nrl)* or unmodified*(Ncl)* in R7E mice.

**Figure 7:**
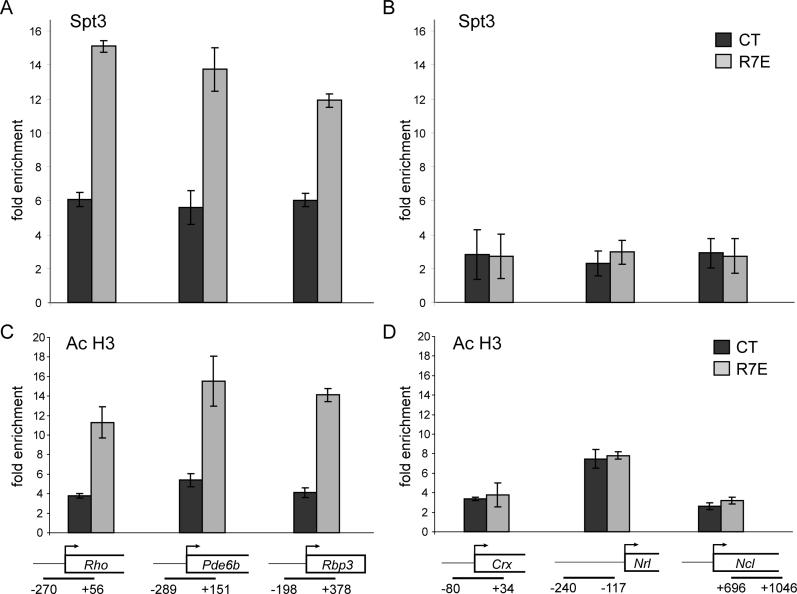
Increased TFTC Recruitment and Histone Acetylation at a Specific Subset of Deregulated Genes in R7E Retina ChIP assays were performed using formaldehyde-fixed chromatin extracts of retina from 2-mo-old control (WT or R7N; dark grey) and R7E (light grey) mice. Primers were selected to amplify promoter or enhancer regions of the specified genes, as depicted. (A and B) ChIPs using an antibody against a TFTC-specific subunit (Spt3) revealed an increased recruitment of Spt3 in R7E retina, at promoters from genes normally highly and specifically expressed in differentiated rods, namely*Rho, Pde6b,* and*Rbp3.* No such differences could be observed at promoters from two genes regulating rod terminal differentiation,*Crx* and*Nrl,* and at the intronic enhancer region of a house-keeping gene,*Ncl.* (C and D) ChIPs using an antibody against acetylated lysines 9 and 14 of histone H3 (Ac H3) revealed an increased acetylation of histone H3 in R7E retina, specifically at the same promoters, which showed increased Spt3 binding*(Rho, Pde6b,* and*Rbp3)*. Promoters from*Crx, Nrl,* and*Ncl* did not show any differences in histone H3 acetylation. The amount of immunoprecipitated DNA was quantified by real-time PCR and normalized to the amount of DNA present in a fraction of the input chromatin extract. Values are expressed as fold enrichment over background IP signals obtained in corresponding no antibody ChIP experiments (seeFigure S9). Each bar represents the mean value ± SEM (*n* = 4).

To monitor TFTC recruitment, we used an antibody raised against SPT3 [31] and found a specific increase (2- to 3-fold) of Spt3 occupancy at the promoters from the*Rho, Pde6b,* and*Rbp3* genes in R7E mice, as compared to control mice (Figure 7A). In contrast, Spt3 recruitment was not modified at the*Crx* and*Nrl* promoters (Figure 7B). Similarly, the housekeeping gene*Ncl* did not show any differences in Spt3 binding (Figure 7B). We next analyzed whether this abnormal recruitment of TFTC-type complexes causes elevated levels of histone modifications at promoters of deregulated genes, possibly accounting for the transcriptional alterations observed in R7E mice. As TFTC preferentially acetylates lysines on the N-terminal tail of histone H3 from 5′ regulatory regions, we quantified H3 K9 and K14 acetylation at the promoter regions previously analyzed. We found an aberrant histone H3 hyperacetylation profile in R7E mice that paralleled aberrant TFTC recruitment.*Rho, Pde6b,* and*Rbp3* promoter regions were hyperacetylated in R7E mice as compared to control mice (Figure 7C). When comparing the ChIP signals obtained at the*Rho, Pde6b,* and*Rbp3* promoters, we observed around a 2- to 3-fold increase both in histone H3 acetylation and in Spt3/TFTC occupancy between R7E and control mice. In contrast, H3 acetylation pattern at promoters from*Crx, Nrl,* and*Ncl* was not modified in R7E mice as compared to control mice (Figure 7D). We also analyzed TFTC-type complexes recruitment at different regions of the*Rho* gene using an anti-SPT3 antibody. We observed a 2-fold increased association of Spt3 at the*Rho* enhancer region, the proximal promoter, and the 3′UTR in R7E mice as compared to control mice (Figure S7). Interestingly, an increased Spt3 occupancy was also observed 2 kb downstream of the*Rho* gene, in R7E animals. Taken together, these results indicate that, in mouse photoreceptors, the polyQ expansion in ATXN7 does not modify its incorporation into TFTC and does not affect its HAT activity. Rather TFTC/STAGA recruitment is deregulated over large genomic regions, leading to histone H3 hyperacetylation, chromatin decondensation, and transcriptional dysregulation.

## Discussion

Based on our findings, we propose that mutant ATXN7, by its incorporation in TFTC-type complexes, deregulates its recruitment, thereby inducing aberrant histone H3 acetylation patterns at specific promoters. Alterations of chromatin-modifying complex regulation likely result in the severe disorganization of the normal chromatin condensation pattern seen specifically in mature rod photoreceptor nuclei. These perturbations eventually cause a loss of the tissue-specific pattern of gene expression, leading to rod photoreceptor dedifferentiation and dysfunction.

### Chromatin Decondensation Leads to Transcriptional Repression of Rod-Specific Genes in SCA7 Mouse Retina

We provide here, to the best of our knowledge, the first evidence that polyQ expansions induce chromatin structure changes in vivo, contributing to early neuronal dysfunction. Taking advantage of the mosaic progression of the R7E retinal phenotype, we showed that*Rho* down-regulation and hence rod dysfunction were completely correlated with chromatin decondensation. Furthermore, we could detect chromatin abnormalities in 4-wk-old R7E mice (Figure S1B), before the onset of an overt retinal dysfunction and concomitantly with a 2-fold*Rho* mRNA decrease [17]. Chromatin decondensation and transcriptional dysregulation of rod-specific genes are thus the earliest molecular abnormalities in R7E mice and occur before retinal dysfunction can be observed. However, transcriptional alterations observed in later stages of R7E retinopathy might additionally result from the severe rod dysfunction that occurs at this age. Candidate gene expression and transcriptome analysis of retina from 2-mo-old R7E mice revealed a severe transcriptional defect affecting rod-specific genes. Accordingly, Bowman et al. [32] suggested that the photoreceptor transcriptional machinery is not globally affected, since rods showing a loss of rhodopsin staining in Sca7^266Q/5Q^ mice had increased Ub^G76V^-GFP transgene mRNA levels. In addition, Yoo et al. [18] reported that several rod-specific genes were downregulated in Sca7^266Q/5Q^ animals. Thus, comparable chromatin and transcriptional abnormalities were observed in both transgenic and knock-in models, suggesting that these observations are relevant for SCA7 retinopathy. Such observations may be also relevant to polyQ toxicity in other neuronal types in other disease models. Identification of the mechanisms underlying specific transcriptional alterations observed in other polyQ diseases [33,34] will help design new therapeutic strategies.

### TFTC/STAGA Dysfunction in SCA7

Using SCA7 mice, we showed here that TFTC/STAGA composition and HAT activity are not affected by incorporation of polyQ-expanded ATXN7. Two recent studies, performed in yeast and in stably transfected 293T embryonic kidney cells, suggested that polyQ-expanded ATXN7 inhibits TFTC/STAGA nucleosomal acetylation activity by interfering with recruitment of SPT3, ADA2b, and TAF12 into these complexes [19,20]. The last two subunits are known as modulators of Gcn5 HAT activity in yeast SAGA [10,30,35,36]. In our study, TFTC/STAGA was immunopurified from SCA7 mouse retina using antibodies against three different subunits. Our results convincingly demonstrate that SCA7 mutant TFTC/STAGA complexes had normal incorporation of Gcn5, Taf12, and Spt3 and, consistently, normal levels of HAT activity on both free histones and nucleosomes. The discrepancies between our in vivo results and previously reported in vitro observations are likely explained by the different model systems used in each study. McMahon et al. analyzed the incorporation of polyQ-expanded human ATXN7 in the yeast SAGA complex. Although yeast Sgf73, Spt3, and Taf12 are the respective homologs of human ATXN7, SPT3, and TAF12, their sequence similarity is low and significant only in one or two conserved domains. In particular, mammalian ATXN7 differs from yeast Sgf73 by the presence of the polyQ tract and an additional third domain that is likely involved in interactions with other TFTC/STAGA subunits [5]. Thus, polyQ-expanded ATXN7 incorporation into yeast SAGA versus human TFTC/STAGA might have different effects on the structure, composition, and function of these complexes. Furthermore, overexpression of other expanded polyQ-containing proteins in yeast has been shown to lead to the formation of intranuclear insoluble aggregates much quicker than in mouse models [37,38]. Thus, in a yeast strain overexpressing mutant ATXN7, its rapid accumulation could alter the available levels of various components of the complex, such as Ada2, Taf12, and Spt3, due to a sequestration or destabilization of these proteins, leading to the quick loss of SAGA HAT activity. Palhan et al. [20] analyzed STAGA complexes from transfected cells using a single antibody against recombinant ATXN7 which could result in the purification of other partial complexes containing overexpressed ATXN7. Furthermore, no transcriptional and/or chromatin alterations were reported in this cell line, making it difficult to correlate impaired HAT activity with SCA7 pathology. In contrast, our results were exclusively obtained from SCA7 mouse retina in which chromatin decondensation is correlated with transcriptional deregulation that likely accounts for the retinal phenotype observed in these mice. In this in vivo model, TFTC/STAGA composition and HAT activity were analyzed before and after the onset of aggregate formation and transcriptional alteration. Finally, using a lymphoblastoid cell line from a SCA7 patient carrying 51 CAG expansion, we previously reported that TFTC composition and HAT activity were unaltered, as compared to control cell lines [39]. In conclusion, these discrepancies demonstrate that in vivo models, which reproduce key features of human pathologies, are likely more significant to understanding disease pathogenesis. However, we cannot exclude that some of our observations in R7E mice might be model specific, and therefore may not account for all pathogenic mechanisms underlying SCA7 retinal phenotype. In light of our observations made on the R7E model, the retina of the SCA7 knock-in mouse model, which recapitulates more accurately SCA7 phenotype in human, will also be further examined, although these mice die quite early, making the analysis difficult.

Previous studies suggested that disruption of the activity of the photoreceptor-specific transcriptional activator Crx may play a role in SCA7 retinopathy by its direct interaction with ATXN7 and that TFTC/STAGA complexes might act as coactivators for Crx-mediated transcriptional activity [20,40,41]. In contrast, other studies showed early and robust transcriptional alterations without modification of Crx levels and activity (Sca7^266Q/5Q^) [18]. Furthermore, expression levels of several genes known to be controlled by Crx were not modified in Sca7^266Q/5Q^ retina whereas other genes not controlled by Crx were found down-regulated [18]. In agreement, we show here a global down-regulation of all rod-specific genes and not exclusively those regulated by Crx, demonstrating that interference of Crx activity does not solely account for the retinal pathogenesis specifically observed in SCA7. Furthermore, we were unable to detect any direct interaction between ATXN7-containing TFTC/STAGA complexes and Crx in WT mouse retina by co-immunoprecipitation experiments (Figure S8). This observation might be explained by the fact that interaction between known activators and co-activators can be temporally and specifically regulated in vivo. Sequential co-ChIP experiments will allow determining whether Crx and TFTC/STAGA can co-occupy promoters from rod-specific genes.

### Involvement of TFTC-Type Complexes in Rod Transcriptional Regulation

Chromatin opening and histone H3 hyperacetylation, normally considered as positive signals for transcription, instead correlated with transcriptional down-regulation of a number of genes in R7E retina, as shown by reduced mRNA levels and decreased RNA Pol II occupancy. Several non-exclusive hypotheses can be proposed to explain how mutant TFTC, containing polyQ-expanded ATXN7, can be aberrantly recruited at certain locations, i.e., at promoters and even nearby intergenic regions, and how this would down-regulate gene expression. One possibility would be that the presence of the polyQ stretch within TFTC serves as an aberrant interaction domain with chromatin, recruiting TFTC at aberrant sites, thus causing histone H3 hyperacetylation and chromatin decondensation, and eventually displacing transcriptional co-activators from their normal localization. Supporting this possibility, large-scale chromatin decondensation has been observed after targeting acidic activators to heterochromatin domains [42,43]. Alternatively, mutant ATXN7 might impair some events downstream of co-activator recruitment, such as the formation of a fully active preinitiation complex containing all the general transcription factors. Indeed, increased occupancy of TFTC complexes did not lead to increased transcription, offering support that additional factors are required to assemble and activate RNA Pol II transcription machinery at rod-specific genes. Finally, mutant TFTC-type complexes may aberrantly acetylate non-histone substrates accounting for chromatin decondensation and directly or indirectly to transcriptional alterations observed in SCA7 mice. Hyperacetylation of HMG proteins or that of histone H1, which was shown to be very efficiently acetylated by TFTC in vitro [6], may be other alternatives to explain the observed chromatin decondensation. HMGI(Y) plays a crucial role for rhodopsin promoter activity [44] and has been shown to be acetylated by PCAF/GCN5 [45].

### Contribution of Chromatin Organization to Rod-Specific Expression Profile

Numerous studies have suggested that changes in nuclear organization establish specific patterns of gene expression, in particular during differentiation [46–49]. Chromatin condensation in rods is tightly regulated because it accompanies rod terminal differentiation and may thus be required for proper regulation of cell type-specific and stage-specific expression in the mouse retina [50] (D. Helmlinger and D. Devys, unpublished data). A recent study suggested a correlation between rod chromatin re-organization and transcriptional repression of rod-specific genes in mice [29]. Together with our observations in R7E mice, this suggests that rod-specific chromatin condensation is required to reach very high expression levels for a specific set of genes encoding proteins required for rod differentiation and function. Our FISH results indicate that in WT rods, a non-expressed gene will be localized in the densely stained central chromatin region, whereas a rod-expressed gene, such as*Rho,* will be localized in the peripheral euchromatin.

Although rod central chromatin displayed histological properties of heterochromatin, little is known about the molecular constituents of this central compact region. It might also contain housekeeping genes with lower expression levels. Rod nuclei might contain chromatin-associated factors, such as histone variants, restricted to mature rods and necessary to regulate the expression of genes involved in rod differentiation and activity. Such proteins were already identified and shown to be required for various developmental processes or during induction/silencing of gene expression [51,52]. Positioning highly expressed genes in the rod-specific euchromatic nuclear periphery might also be used to accelerate the coupling between mRNA synthesis, processing, and export, thereby accelerating the total rate of protein synthesis [53,54].

These findings might explain why such a massive chromatin remodeling in SCA7 mice affected only those genes whose expression and nuclear distribution are the most tightly regulated. These genes (i.e.,*Rho, Pde6b,* and*Rbp3*) are characterized by an increased TFTC-binding, histone H3 hyperacetylation, but surprisingly with a transcriptional decrease in SCA7 mouse retina. We hypothesize that at the corresponding promoters, TFTC requirement is normally highly regulated and that aberrant, non-specific TFTC recruitment over a large genomic region (Figure S7) causes deregulated Pol II transcription. These highly expressed genes may be particularly sensitive to the changes occurring in the chromatin territories and to the consequent dilution of general transcription factors in the increased nuclear volume. In contrast, expression of a majority of genes is not changing despite severe chromatin reorganization, because these genes are probably weakly transcribed and thus are not sensitive to the changes in nuclear environment.

Altogether our study may provide new insights into SCA7 pathogenesis. Deciphering the exact causal relationships between TFTC/STAGA aberrant recruitment, histone hyperacetylation, and chromatin decondensation will require further investigation. These future directions should shed light on the transcriptional mechanisms initiating and controlling a tissue-specific gene expression pattern and the contribution of TFTC-type coactivator complexes to such processes in vivo.

## Materials and Methods

### Animals

R7N and R7E animals were from the R7N.C and R7E.A transgenic lines, respectively, and were maintained on the inbred C57BL/6 background [16]. The Sca7^266Q/5Q^ C1 line was backcrossed with C57Bl/6J [18]. Genotyping was performed using PCR on mouse tail genomic DNA according to procedures described previously [16,18]. All experiments were performed in accordance with the National Institutes of Health*Guide for the Care and Use of Laboratory Animals*.

### Quantitative RT-PCR analysis of retinal RNA

We prepared total RNA by single-step extraction of guanidium thiocyanate retinal homogenates. Reverse transcription was performed on 1 μg total RNA using SuperScriptII (Invitrogen, Carlsbad, California, United States) and random hexamers according to the manufacturer's instructions. PCR amplifications of cDNA were performed using SYBR Green I (Sigma-Aldrich, St Louis, Missouri, United States) on a LightCycler instrument (Roche Products, Basel, Switzerland). PCR primers were designed using Primer3 software (http://frodo.wi.mit.edu/primer3/primer3_code.html) and are available upon request.

### IP and histone acetylation assay

Extracts from two retinas are immunoprecipitated using 50 μl of protein A-Sepharose beads and polyclonal antibodies (anti-TRRAP 1930 or anti-ATXN7 1261 as described [5]) or monoclonal antibodies for SPT3. After washing IP, beads are immediately boiled and processed for Western blot analysis or used for a HAT assay. The HAT test was performed on free histones or on nucleosomes as described [6]. A list of used antibodies is available inTable S2.

### Electron microscopy, histology, and immunohistology

For light and electron microscopy, enucleated eyes were fixed by immersion in 2.5% glutaraldehyde in PBS 1× for 16 h at 4 °C. The lens and cornea were removed. After rinsing in PBS, the eyes were postfixed in 1% osmium tetroxide in the same buffer for 2 h at 4 °C, dehydrated with graded alcohol series, and embedded in Epon. For light microscopy, semi-thin sections (1 μm) were stained with toluidine blue. Ultrathin sections were contrasted with uranyl acetate and lead citrate, and examined with a Philips 208 electron microscope (Philips, Best, Netherlands) operating at 60 kV. For immunohistochemistry and immunohistofluorescence, enucleated eyes were dissected to remove lens and cornea, and fixed by immersion in 4% paraformaldehyde for 2 h, 0.3 mM CaCl_2_, 0.1 mM MgCl_2_, 1× PBS. For immunochemistry, fixed eyecups were dehydrated, embedded in paraffin, and cut into 5-μm sections. For immunofluorescence, fixed eyecups were cryoprotected in 30% sucrose, frozen and embedded in Cryomatrix (Thermo Shandon, Pittsburgh, Pennsylvania, United States), and then cut into 10 μm cryostat sections mounted on SuperFrost/Plus slides (O. Kindler, Freiburg, Germany). Sections were permeabilized and blocked for 1 h with 10% normal goat serum, 0.5% bovine serum albumin, 0.1% Tween 20, and 1× PBS. For immunochemistry, biotinylated goat antirabbit IgG (Vector Laboratories, Burlingame, California, United States), streptavidin-biotin complex solution (Vector Laboratories), and diaminobenzidine (Vector Laboratories) were used according to the instructions of the manufacturer. Sections were not counterstained to better contrast the DAB staining. For immunofluorescence, secondary antibodies used were CY3- and Oregon Green-conjugated goat anti-rabbit and anti-mouse IgG (Jackson ImmunoResearch, West Grove, Pennsylvania, United States), and nuclei were counterstained with 0.5 μg/ml DAPI (4,6-diaminido-2-phenylindole).

### FISH

BAC clones were obtained from BACPAC Resources Center (BPRC, Oakland, California, United States), biotin-labeled by nick translation, and used as DNA FISH probes. We used BAC clones RP24-248E16 for*Rho* and RP24-329P13 for*Acta2*. The specificity of labeled probes was checked by FISH analysis of mouse metaphase spreads that were prepared and hybridized with 200 ng of each probe per slide, according to standard methods.

We then developed a protocol for interphase FISH of mouse retina, based on a previously described procedure [55]. Indeed, we noticed that standard pre-treatment conditions altered rod nuclear morphology, as revealed by DAPI staining, possibly modifying the localization of*Rho* and*Acta2.* Different slide pretreatment conditions were applied in order to improve the hybridization signal-to-noise ratio without completely altering rod nuclear architecture. Paraffin-embedded mouse retinal sections (5 μm) were prepared as for immunohistochemistry. Slides were deparaffinized with xylene, hydrated with a series of graded ethanol, and permeabilized in 0.1% Triton-X 100 at room temperature for 5 min. Slides were then treated either with 1M NaSCN at 80 °C for 30 min or with 20% or 50% glycerol at 80 °C for 30 min. For digestion, approximately 100 μg proteinase K (10 mg/ml in 2× SSC; Sigma) per slide was applied and incubated at 37 °C for 45 min. Slides were then washed twice in 2× SSC for 2 min, post-fixed in 1% formaldehyde (in 1× PBS/50 mM MgCl_2_) for 10 min at room temperature, rinsed in 1× PBS for 5 min, and then dehydrated with a series of graded ethanol. Denaturation was realized in 70% formamide (in 2× SSC) at 80 °C for 3 min. Dehydration was then carried out with a series of cold (−20 °C) graded ethanol, and slides were air dried. Meanwhile, labeled DNA probes were precipitated with 10 μg Cot-1 DNA (1 mg/ml; Invitrogen) and 1.5 μg ssDNA (10 mg/ml; Sigma) per slide. Probes were first resuspended in 70% deionized formamide (in 2× SSC) and then in 20% dextran sulphate (in 4× SSC) at 37 °C for at least 30 min. DNA probes were then denatured at 76 °C for 5 min, pre-annealed at 37 °C for at least 1 h, applied to denatured slides, and hybridized by overnight incubation at 37 °C. Post-hybridization was then performed according to standard procedures. Briefly, slides were washed three times for 5 min each in 50% formamide, 2× SSC at 42 °C, and three times for 5 min each in 0.5× SSC at 42 °C. Slides were then permeabilized in 0.1% Tween-20 (4× SSC) for 5 min at room temperature, blocked in 3% BSA for 30 min at 37 °C, and sequentially treated with FITC (fluorescein-labeled ) streptavidin (Vector), biotin-streptavidin (Vector), and FITC-strepatavidin, in 1% BSA/0.1% Tween-20/4× SSC, each for 30 min at 37 °C. After washing, slides were incubated with 0.5 μg/ml DAPI, dehydrated in a graded series of ethanol, air dried, and mounted in Vectashield (Vector). Nuclei and FISH signals were visualized and acquired by confocal microscopy. DAPI fluorescence was pseudo-colored using confocal specific filters.

### ChIP

Dissected retinas were immediately incubated for 10 min at room temperature in 1% formaldehyde with rocking. Cross-linking was stopped by the addition of 0.125M glycine for 5 min at room temperature, and retinas were washed in PBS containing 1 mM phenylmethylsulfonyl fluoride (PMSF) and 1× protease inhibitors cocktail (PIC; Sigma). Two retinas (one mouse) were pooled and homogenized on ice in 500 μl lysis buffer (20 mM HEPES-KOH [pH 8.0], 10 mM KCl, 0.1% NP-40, 1 mM PMSF, and PIC) using a Dounce homogenizer (pestle B; Kimble/Kontes, Vineland, New Jersey, United States). Nuclei were recovered by table-top centrifugation (5 min at 5,000 rpm, 4 °C), resuspended in 120 μl sonication buffer (50 mM HEPES-KOH [pH 8.0], 140 mM NaCl, 1 mM EDTA, 1% Triton-X 100, 0.1% NaDOC, 0.1% SDS, 1 mM PMSF, and PIC) and incubated on ice for 10 min. Cross-linked chromatin extracts were then diluted 4.5-fold in IP buffer (20 mM Tris-HCl [pH 8.0], 100 mM NaCl, 2 mM EDTA, 0.5% Triton-X 100, 1 mM PMSF, and PIC) and then sonicated to an average DNA length of 400-1000 base pairs (Figure S9). After centrifugation (at 4 °C for 15 min at full speed), 40 μl of the supernatants were used as inputs and the remainder (∼1.5 mg, i.e., ∼1.3 × 10^7^ rod cells from two whole retina) used for one IP. Extracts were first pre-cleared with 20-μl preimmune IgG, 200-μg salmon sperm DNA, 0.1% BSA, and 50-μl protein A- or G-Sepharose beads (Sigma) slurry for 2 h at 4 °C. These beads were prepared by two washings in PBS and resuspension in 1 mM EDTA, 10 mM Tris-HCl (pH 8.1). Pre-cleared extracts were incubated with 2 to 5 μg of anti-RNA Pol II CTD (7G5), anti-SPT3 (2C10), anti-acetyl K9, K14 H3 antibody (06–599; Upstate Biotechnology, Charlottesville, Virginia, United States) or the appropriate amount of rabbit preimmune serum as control, for 16 h at 4 °C. Immune complexes were recovered by a 5-h incubation at 4 °C with protein A- or G-Sepharose. Beads were serially washed with 500-μl low-salt buffer (20 mM Tris-HCl [pH 8.0], 150 mM NaCl, 2 mM EDTA, 1% Triton-X 100, 0.1% SDS, 1 mM PMSF, and PIC), high-salt buffer (same as before with 500 mM NaCl), lithium chloride buffer (10 mM Tris-HCl [pH 8.0], 250 mM LiCl, 1 mM EDTA, 1% NaDOC, 1% NP-40, 1 mM PMSF, and PIC), and twice in 1 mM EDTA, 10 mM Tris-HCl (pH 8.0). Immunoprecipitated chromatin complexes were eluted twice from beads with 1% SDS, 0.1 mM NaHCO_3_, each time for 30 min at 65 °C, with vortexing each 5 min. Cross-linking was reversed by overnight incubation at 65 °C, with 10 μg RNase A in 200 mM NaCl. DNA was then recovered by digestion with 20 μg of proteinase K in 5 mM EDTA for 2 h at 42 °C and was purified by phenol-chloroform extraction. DNA sequences present in the immunoprecipitates were quantified by real-time PCR using SYBR Green I (Sigma-Aldrich) on a LightCycler instrument (Roche). All PCR primers were designed using Primer3 software and are available upon request. DNA amounts were calculated by comparison to a standard curve generated by serial dilutions of input DNA. Input represented 4% of the total amount of chromatin used for each IP sample (2 retinas). Fold enrichment for each antibody was calculated as the ratio of immunoprecipitated sequence over total sequence amount in input chromatin and normalized to the ratio obtained without antibody (Figure S9A).

## Supporting Information

Figure S1 Chromatin Decondensation in R7E Mice (A) Localization of normal and mutant ataxin-7 in rod nuclei from R7N or R7E mice, respectively. Retinal cryosections (10 μm) from 2-y-old R7N and R7E animals were stained using anti-ATXN7 antibody (1261, green) and DAPI (pseudocolored in red) to visualize rod nuclei. Merge images revealed normal ATXN7 localization in the thin rim of peripheral euchromatin in rod nuclei. Mutant ATXN7 is aggregated into a single, large NI within the decondensed chromatin, adjacent to the remaining central heterochromatin territory. Scale bar represents 3 μm.
(B) Chromatin decondensation is detected before the onset of R7E retinopathy. Histological examination of retina from 4-wk-old R7N (left panel) and R7E (middle and right panels) mice. Toluidin-blue staining of semi-thin sections revealed a mild decondensation of rod chromatin in R7E animals, predominantly in the outer part of the outer nuclear layer (ONL).
(162 KB PDF).

Figure S2 Electron Microscopy of 2-Y-Old R7E Retina (A) Note the almost complete absence of outer and inner segments (OS + IS) because photoreceptor nuclei (ONL) are in close proximity to the retinal pigmented epithelium (RPE).
(B–C) In both WT and R7E mice, cells from the retinal pigment epithelium displayed numerous apical microvilli contacting photoreceptor segments and showed typical outer segment phagocytosis (PH) [56]. These observations further indicate that rod outer segment loss in R7E mice is due to a defect in their renewal.
Scale bars represent 3 μm in (A) and (B) and 0.5 μm in (C).
av, apical microvilli; bm, Bruch's basement membrane; IS, inner segments; m, melanin pigments; n, nuclei of RPE cells; olm, outer limiting membrane; OS, outer segments; PH, phagosomes; pn, photoreceptor nuclei; pos, photoreceptor outer segments; RPE, retinal pigment epithelium.
(445 KB PDF).

Figure S3 Ultrastructure of NIs Found in Rod Photoreceptors from 3-mo-Old Sca7^266Q/5Q^ Mice Examples of NIs (indicated by large arrows in a and c) found within rods by conventional electron microscopy. At higher magnifications, the NI appeared as a pale-stained structure surrounded by euchromatin (eu), distinct from the darkly stained adjacent heterochromatin (h) territory and central nucleolus (Nu). Scale bars repesent 1 μm in a, 0.25 μm in b, d, and e, and 0.5 μm in c.
eu, euchromatin; h, heterochromatin; nu, nucleolus.
(400 KB PDF).

Figure S4 Unique Architecture of Rod Photoreceptor Nuclei Retinal cryosections (10 μm) from 2-mo-old WT animals were stained using DAPI (blue) and antibodies against various nuclear proteins (red): RNA polymerase II (A), TBP (B), a TFTC/STAGA subunit ATXN7 (C), unmodified histone H3 (D), K9, K14 acetylated histone H3 (E), the photoreceptor specific transcriptional activator Crx (F), the heterochromatin protein HP1γ (G), the non-histone chromosomal protein HMGI(Y) (H), and a transcriptional repressor (TIF1β) (I). Fluorescence images were collected and merged to visualize the intranuclear localization of several transcription factors and chromatin regulators in WT rod nuclei. All proteins analyzed present a characteristic ring-like appearance and are predominantly localized to the thin rim of peripheral euchromatin. Scale bar represents 5 μm.
(150 KB PDF).

Figure S5 Demonstration of FISH Probes Specificity FISH of*Rho* and*Acta2* probes to mouse embryonic stem cell spreads revealed specific hybridization on metaphase chromosomes and interphase nuclei, counterstained with DAPI. Scale bar represents 5 μm.
(77 KB PDF).

Figure S6 TFTC Histone Acetyl Transferase Activity on Histone or Nucleosomal Substrates Is Not Altered by ATXN7 PolyQ Expansion in R7E Retina (A) Quantification of the HAT activities of TFTC-type complexes immunopurified from R7N and R7E retina. HAT assays were performed after immunopurifications using anti-ATXN7, anti-SPT3, or anti-TRRAP antibodies, as indicated inFigure 6. No significant differences could be detected when comparing R7N and R7E mice. Each HAT activity was calculated and is represented as a ratio to the mean of all animals analyzed in each assay. Each bar represent the mean value ± the standard error of the mean (*n* = 2–7).
(B–C) HAT activities of immunopurified complexes bound to the anti-SPT3 monoclonal antibody (mAb) (B) or anti-TRRAP mAb (C) beads from R7N and R7E retina were measured by an in vitro HAT assay performed on oligonucleosomes prepared from HeLa cells. Histones were separated by SDS-PAGE, and acetylated histones were visualized by fluorography (Fluorgr). The position of each histone is indicated. The age of the mice are indicated in weeks in brackets.
(146 KB PDF).

Figure S7 Increased Recruitment of TFTC-Type Complexes Containing PolyQ-Expanded ATXN7 at Different Regions of the*Rho* Gene ChIP assays were performed using formaldehyde-fixed chromatin extracts of retina from 2-mo-old control (WT or R7N; dark grey) and R7E (light grey) mice. Specific regions within the*Rho* gene were analyzed by real-time PCR, after ChIP using an antibody against a TFTC-specific subunit (Spt3). Quantification of fold enrichment was calculated as indicated inFigure S9. Each bar represents the mean value ± the standard error of the mean (*n* = 4–6).
(73 KB PDF).

Figure S8 Absence of In Vivo Interaction of Crx with TFTC/STAGA Complexes (A) Whole retinal extracts from WT or normal ATXN7-expressing mice (R7N) were immunoprecipitated with two distinct anti-Trrap polyclonal antibodies (lanes 2 and 4) or one anti-ATXN7 monoclonal antibody (lane 6). Immunoprecipitated complexes retained on protein A-Sepharose beads were analyzed by immunoblotting with a polyclonal antibody detecting Crx. Similar experiments were performed using the anti-Crx polyclonal antibody for IP and a monoclonal anti-ATXN7 antibody for immunoblot analysis. Both panels demonstrated absence of co-immunoprecipitation of Crx with either Trrap or ATXN7 in mouse retina (lanes 2, 4, 6, and 8). IgG bands were detected with the secondary anti-rabbit peroxidase-conjugated antibodies (lanes 2 and 4). Input (lanes 1, 3, 5, and 7) represents 10% of the amount of whole retinal extracts used for IP.
(1.2 MB PDF).

Figure S9 ChIP Experiment Design (A) Quantification of the fold enrichment for each antibody at specific loci was obtained by calculating the ratio of immunoprecipitated DNA relative to input DNA amounts, normalized to the ratio obtained with a control IP (no antibody).
(B) Representative example of sonicated input chromatin isolated from mouse retina (seeMaterials and Methods).
(74 KB PDF).

Table S1 Comprehensive List of Deregulated Genes in Retina from 2-mo-Old R7E Mice (51 KB DOC).

Table S2 Source of Antibodies Used and Working Dilutions (70 KB DOC).

## Abbreviations

ChIP: chromatin immunoprecipitation
FISH: fluorescent in situ hybridization
HAT: histone acetyltransferase
IP: immunoprecipitation
mAb: monoclonal antibody
NI: nuclear inclusion
ONL: outer nuclear layer
polyQ: polyglutamine
SCA7: spinocerebellar ataxia type 7
SEM: standard error of the mean
TAF: TBP-associated factor
TBP: TATA-binding protein
WT: wild-type
