# Supplementary material for: Glutamine-Expanded Ataxin-7 Alters TFTC/STAGA Recruitment and Chromatin Structure Leading to Photoreceptor Dysfunction
Source: PLoS Biol. 2006 Feb 28;4(3):e67. doi: 10.1371/journal.pbio.0040067 (PMC1382020; doi:10.1371/journal.pbio.0040067)
Supplement: Supplementary file 1 [file pbio.0040067.sg001.pdf]

SUPPORTING INFORMATION: 9 Figures

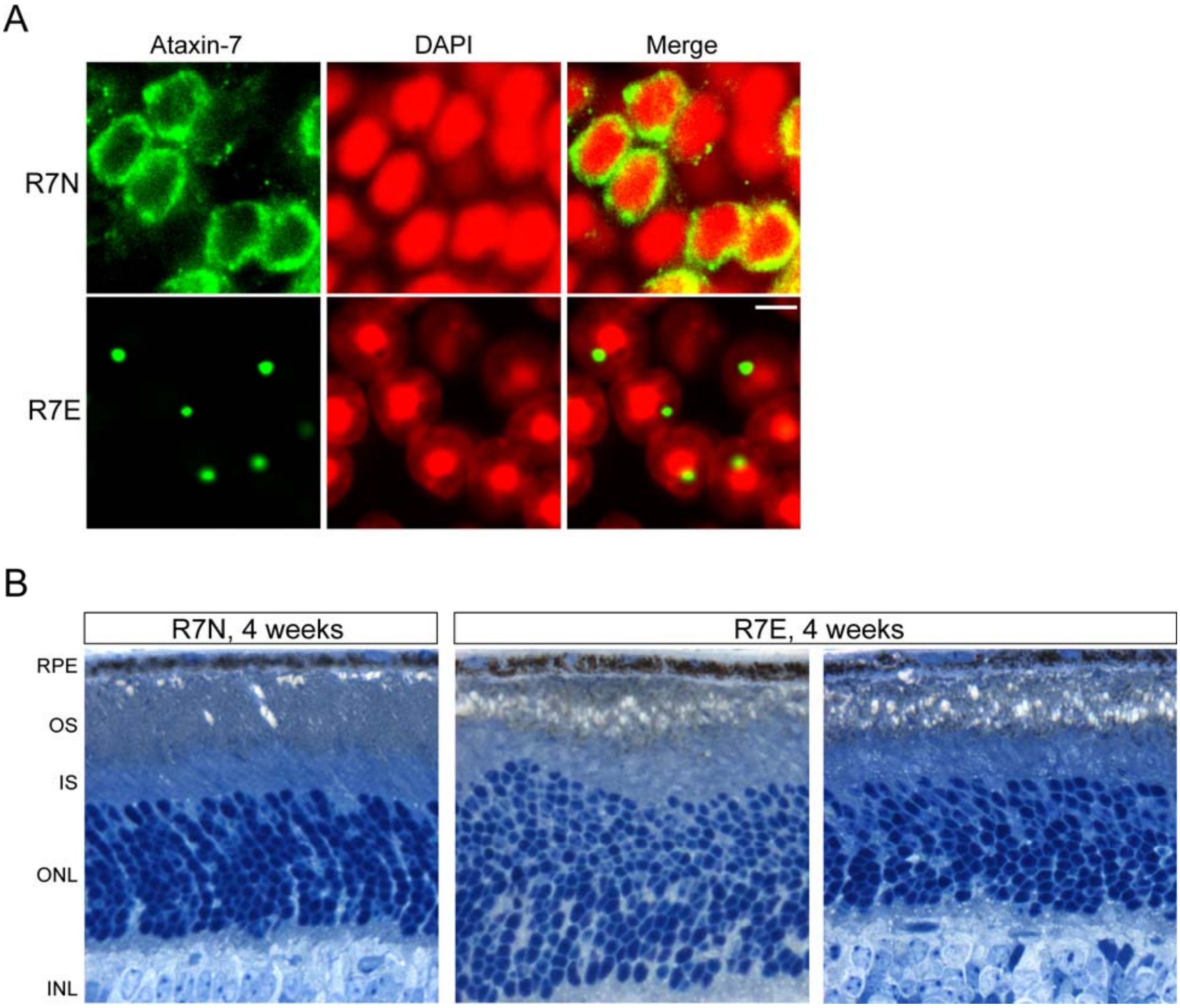

Figure S1: Chromatin decondensation in R7E mice.

A. Localization of normal and mutant ataxin-7 in rod nuclei from R7N or R7E mice, respectively. Retinal cryosections (10 µm) from 2-year-old R7N and R7E animals were stained using anti-ATXN7 antibody (1261, green) and DAPI (pseudocolored in red) to visualize rod nuclei. Merge images revealed normal ATXN7 localization in the thin rim of peripheral euchromatin in rod nuclei. Mutant ATXN7 is aggregated into a single, large nuclear inclusion within the decondensed chromatin, adjacent to the remaining central heterochromatin territory. Scale bar represents 3 µm.

B. Chromatin decondensation is detected before the onset of R7E retinopathy. Histological examination of retina from 4 week-old R7N (left panel) and R7E (middle and right panels) mice. Toluidin-blue staining of semi-thin sections revealed a mild decondensation of rod chromatin in R7E animals, predominantly in the outer part of the ONL.
