# Supplementary material for: Glutamine-Expanded Ataxin-7 Alters TFTC/STAGA Recruitment and Chromatin Structure Leading to Photoreceptor Dysfunction
Source: PLoS Biol. 2006 Feb 28;4(3):e67. doi: 10.1371/journal.pbio.0040067 (PMC1382020; doi:10.1371/journal.pbio.0040067)
Supplement: Supplementary file 2 [file pbio.0040067.sg002.pdf]

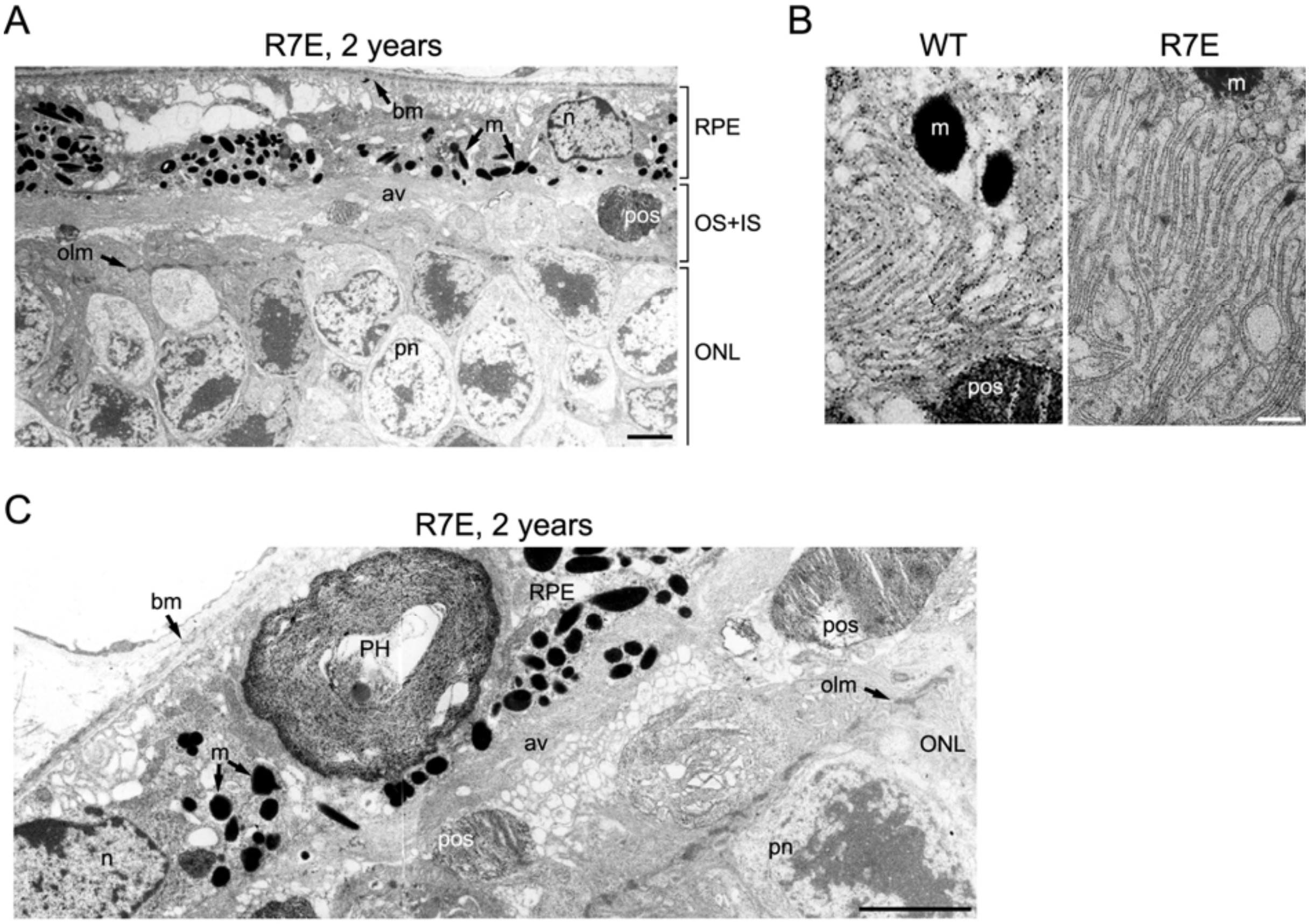

Figure S2: Electron microscopy of 2-year-old R7E retina.

A. Note the almost complete absence of outer and inner segments (OS + IS) as photoreceptor nuclei (ONL) are in close proximity to the retinal pigmented epithelium (RPE).

B-C. In both wild-type and R7E mice, cells from the retinal pigment epithelium displayed numerous apical microvilli contacting photoreceptor segments and showed typical outer segment phagocytosis (PH). These observations further indicate that rod outer segment loss in R7E mice is due to a defect in their renewal. Scale bars are 3 µm in A and B and 0.5 µm in C. Abbreviations: av, apical microvilli; bm, Bruch’s basement membrane; IS, inner segments; m, melanin pigments; n, nuclei of RPE cells; olm, outer limiting membrane; OS, outer segments; ONL, outer nuclear layer; PH, phagosomes; pos, photoreceptor outer segments; pn, photoreceptor nuclei; RPE, retinal pigment epithelium.
