# Supplementary material for: Glutamine-Expanded Ataxin-7 Alters TFTC/STAGA Recruitment and Chromatin Structure Leading to Photoreceptor Dysfunction
Source: PLoS Biol. 2006 Feb 28;4(3):e67. doi: 10.1371/journal.pbio.0040067 (PMC1382020; doi:10.1371/journal.pbio.0040067)
Supplement: Supplementary file 3 [file pbio.0040067.sg003.pdf]

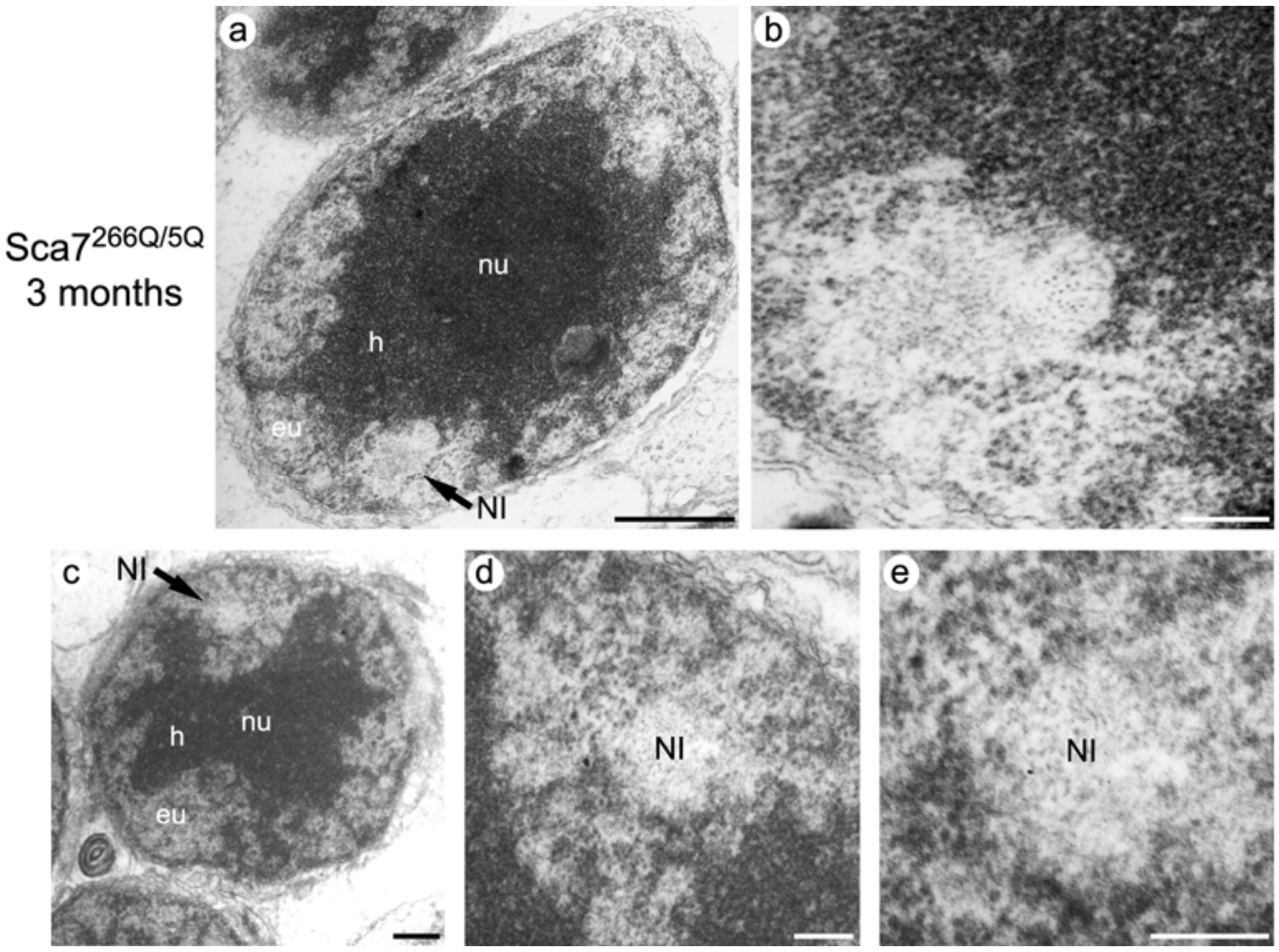

Figure S3: Ultrastructure of NIs found in rod photoreceptors from 3-month-old Sca7$^{266Q/5Q}$ mice.

Examples of nuclear inclusions (NI, indicated by large arrows in a and c) found within rods by conventional electron microscopy. At higher magnifications, the NI appeared as a pale stained structure surrounded by euchromatin (eu), distinct from the darkly stained adjacent heterochromatin (h) territory and central nucleolus (Nu). Scale bars are 1 µm in a, 0.25 µm in b, d, e and 0.5 µm in c. Abbreviations: eu, euchromatin; h, heterochromatin; NI, nuclear inclusion; nu, nucleolus.
