# Supplementary material for: Glutamine-Expanded Ataxin-7 Alters TFTC/STAGA Recruitment and Chromatin Structure Leading to Photoreceptor Dysfunction
Source: PLoS Biol. 2006 Feb 28;4(3):e67. doi: 10.1371/journal.pbio.0040067 (PMC1382020; doi:10.1371/journal.pbio.0040067)
Supplement: Supplementary file 4 [file pbio.0040067.sg004.pdf]

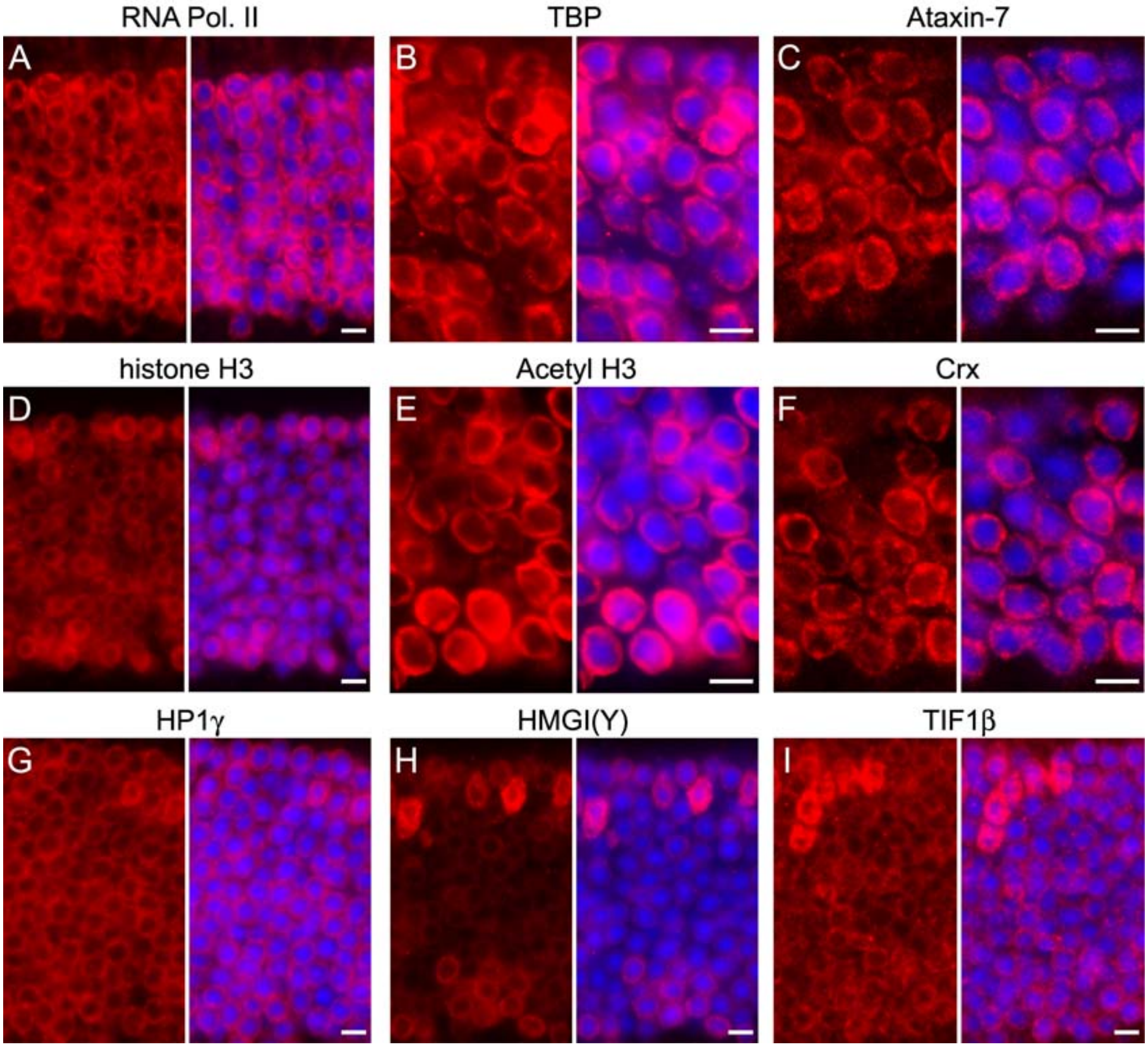

Figure S4: Unique architecture of rod photoreceptor nuclei.

Retinal cryosections (10 µm) from 2-month-old WT animals were stained using DAPI (blue) and antibodies against various nuclear proteins (red): RNA polymerase II (A), TBP (B), a TFTC/STAGA subunit ATXN7 (C), unmodified histone H3 (D), K9, K14 acetylated histone H3 (E), the photoreceptor specific transcriptional activator Crx (F), the heterochromatin protein HP1γ (G), the non-histone chromosomal protein HMGI(Y) (H) and a transcriptional repressor (TIF1β). Fluorescence images were collected and merged to visualize the intranuclear localization of several transcription factors and chromatin regulators in wild-type rod nuclei. All proteins analyzed present a characteristic ring-like appearance and are predominantly localized to the thin rim of peripheral euchromatin. Scale bar represents 5 µm.
