# Supplementary material for: Glutamine-Expanded Ataxin-7 Alters TFTC/STAGA Recruitment and Chromatin Structure Leading to Photoreceptor Dysfunction
Source: PLoS Biol. 2006 Feb 28;4(3):e67. doi: 10.1371/journal.pbio.0040067 (PMC1382020; doi:10.1371/journal.pbio.0040067)
Supplement: Supplementary file 5 [file pbio.0040067.sg005.pdf]

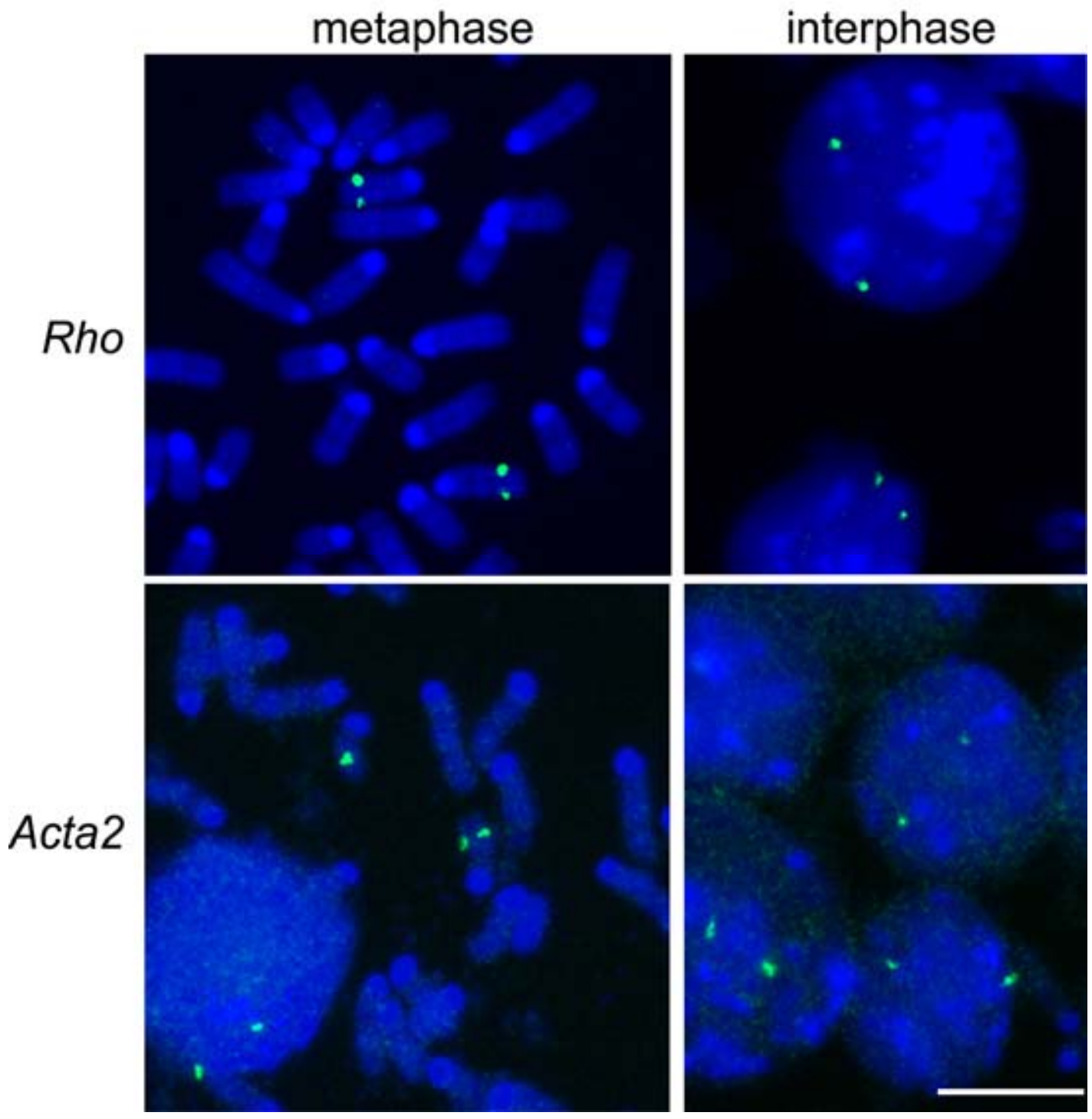

Figure S5: Demonstration of FISH probes specificity.

FISH of *Rho* and *Acta2* probes to mouse ES cell spreads revealed specific hybridization on metaphase chromosomes and interphase nuclei, counterstained with DAPI. Scale bar is 5 μm.
