# Supplementary material for: Glutamine-Expanded Ataxin-7 Alters TFTC/STAGA Recruitment and Chromatin Structure Leading to Photoreceptor Dysfunction
Source: PLoS Biol. 2006 Feb 28;4(3):e67. doi: 10.1371/journal.pbio.0040067 (PMC1382020; doi:10.1371/journal.pbio.0040067)
Supplement: Supplementary file 6 [file pbio.0040067.sg006.pdf]

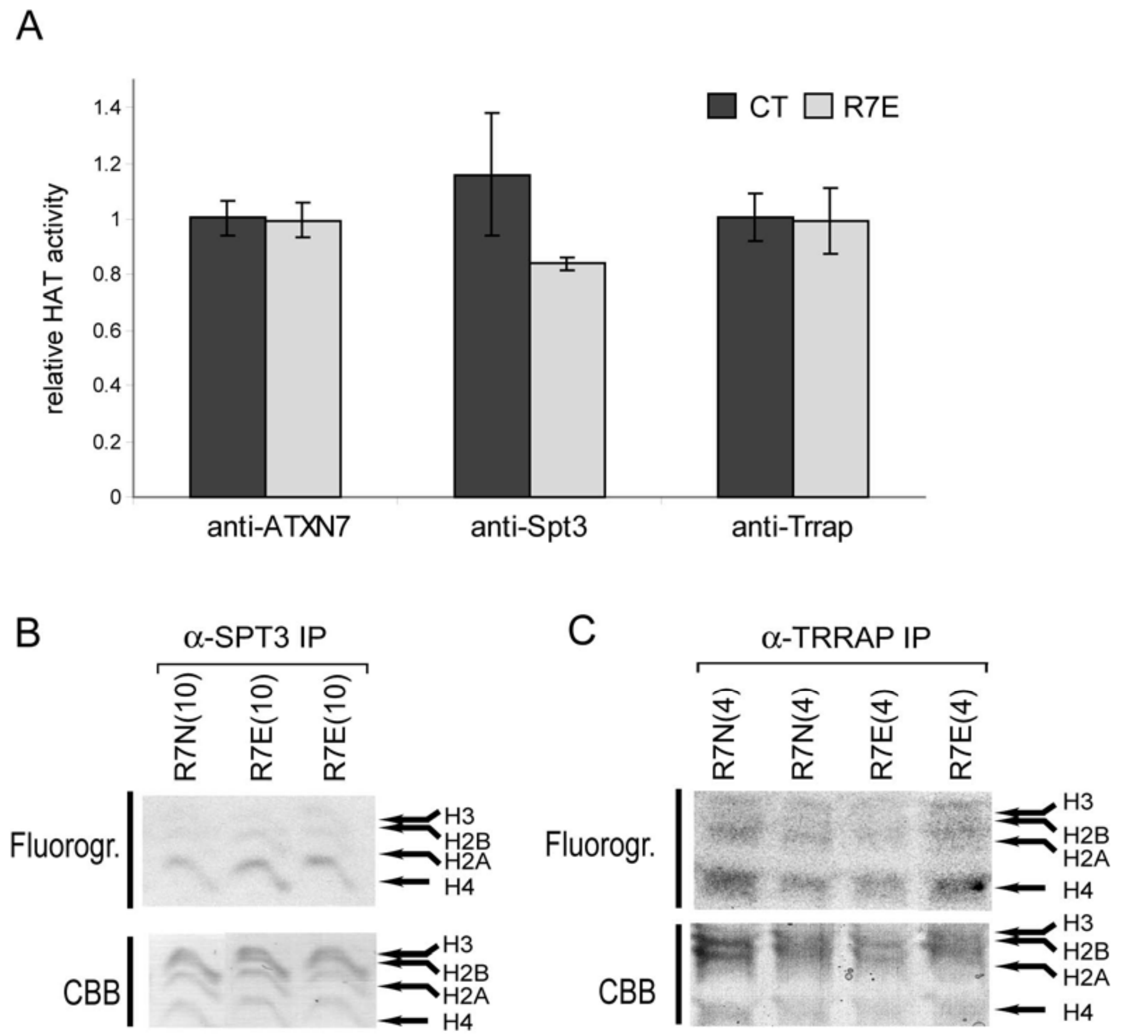

Figure S6: TFTC histone acetyl transferase activity on histone or nucleosomal substrates is not altered by ATXN7 polyglutamine expansion in R7E retina.

A. Quantification of the HAT activities of TFTC-type complexes immunopurified from R7N and R7E retina. HAT assays were performed after immunopurifications using anti-ATXN7, anti-SPT3 or anti-TRRAP antibodies, as indicated in Figure 6. No significant differences could be detected when comparing R7N and R7E mice. Each HAT activity was calculated and is represented as a ratio to the mean of all animals analyzed in each assay. Each bar represent the mean value ± SEM (n = 2-7).

B-C. HAT activities of immunopurified complexes bound to the anti-SPT3 mAb (B) or anti-TRRAP mAb (C) beads from R7N and R7E retina were measured by an *in vitro* HAT assay performed on oligonucleosomes prepared from HeLa cells. Histones were separated by SDS-

PAGE and acetylated histones were visualized by fluorography (Fluorgr.). The position of each histone is indicated. The age of the mice are indicated in weeks in brackets.
