# Supplementary material for: Glutamine-Expanded Ataxin-7 Alters TFTC/STAGA Recruitment and Chromatin Structure Leading to Photoreceptor Dysfunction
Source: PLoS Biol. 2006 Feb 28;4(3):e67. doi: 10.1371/journal.pbio.0040067 (PMC1382020; doi:10.1371/journal.pbio.0040067)
Supplement: Supplementary file 7 [file pbio.0040067.sg007.pdf]

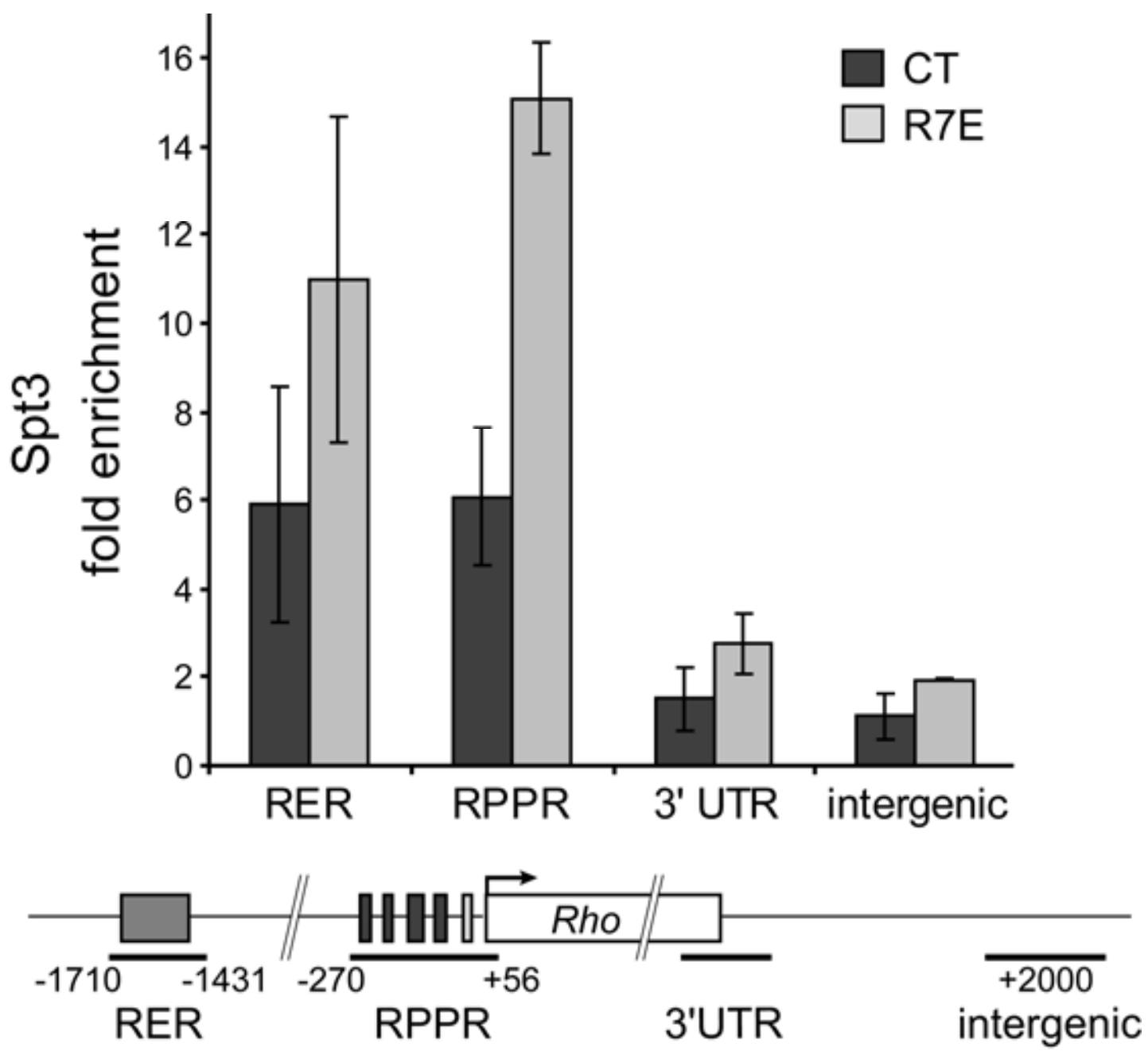

Figure S7: Increased recruitment of TFTC-type complexes containing polyglutamine expanded ATXN7 at different regions of the *Rho* gene.

Chromatin immunoprecipitation assays (ChIP) were performed using formaldehyde-fixed chromatin extracts of retina from 2 month-old control (WT or R7N; dark grey) and R7E (light grey) mice. Specific regions within the *Rho* gene were analyzed by real-time PCR, after ChIP using an antibody against a TFTC-specific subunit (Spt3). Quantification of fold enrichment was calculated as indicated in Figure S9. Each bar represents the mean value ± SEM (n = 4-6).
