# Supplementary material for: Glutamine-Expanded Ataxin-7 Alters TFTC/STAGA Recruitment and Chromatin Structure Leading to Photoreceptor Dysfunction
Source: PLoS Biol. 2006 Feb 28;4(3):e67. doi: 10.1371/journal.pbio.0040067 (PMC1382020; doi:10.1371/journal.pbio.0040067)
Supplement: Supplementary file 8 [file pbio.0040067.sg008.pdf]

**SUPPORTING INFORMATION: 9 Figures**

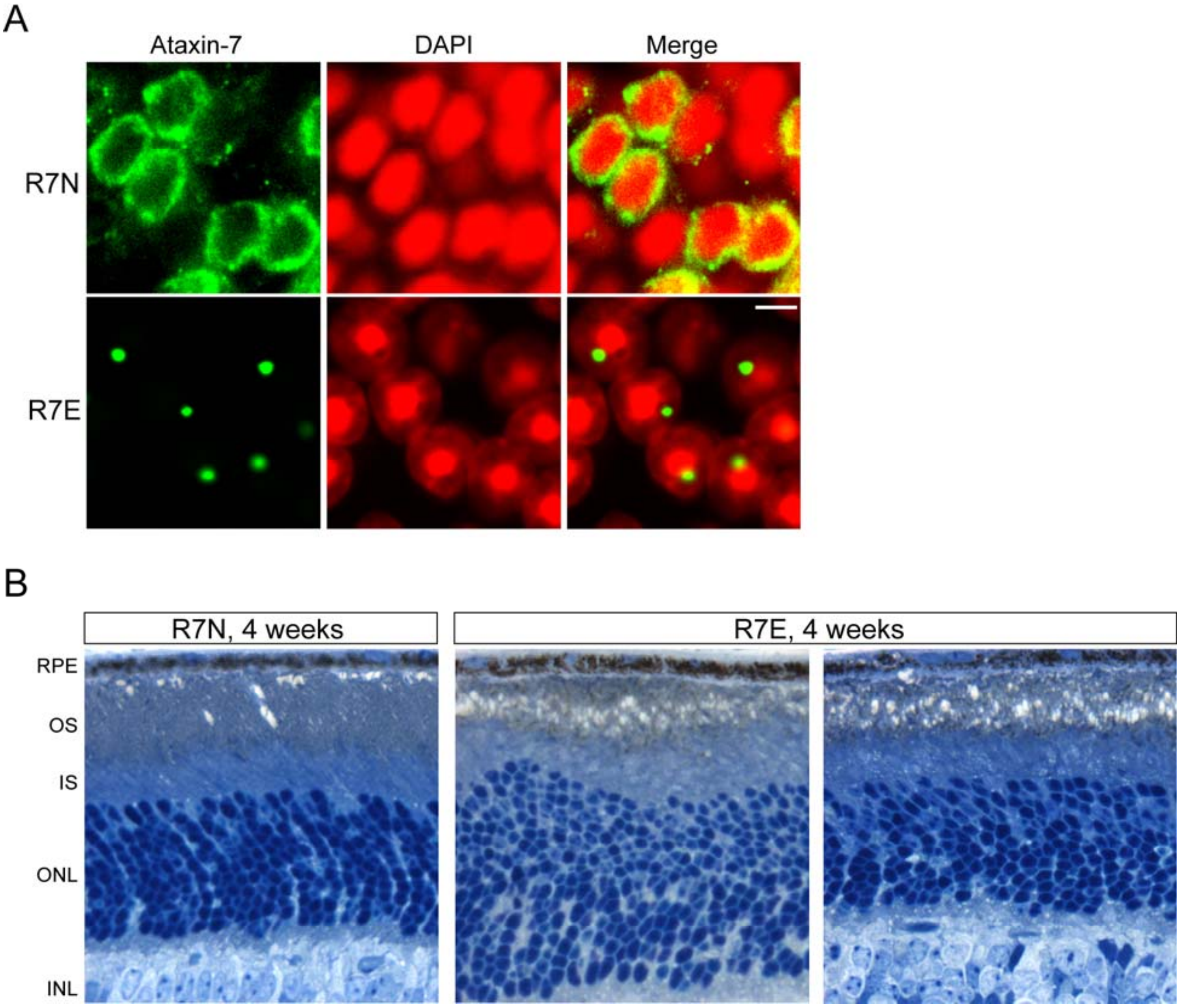


**Figure S1: Chromatin decondensation in R7E mice.**

A. Localization of normal and mutant ataxin-7 in rod nuclei from R7N or R7E mice, respectively. Retinal cryosections (10 µm) from 2-year-old R7N and R7E animals were stained using anti-ATXN7 antibody (1261, green) and DAPI (pseudocolored in red) to visualize rod nuclei. Merge images revealed normal ATXN7 localization in the thin rim of peripheral euchromatin in rod nuclei. Mutant ATXN7 is aggregated into a single, large nuclear inclusion within the decondensed chromatin, adjacent to the remaining central heterochromatin territory. Scale bar represents 3 µm.

B. Chromatin decondensation is detected before the onset of R7E retinopathy. Histological examination of retina from 4 week-old R7N (left panel) and R7E (middle and right panels) mice. Toluidin-blue staining of semi-thin sections revealed a mild decondensation of rod chromatin in R7E animals, predominantly in the outer part of the ONL.

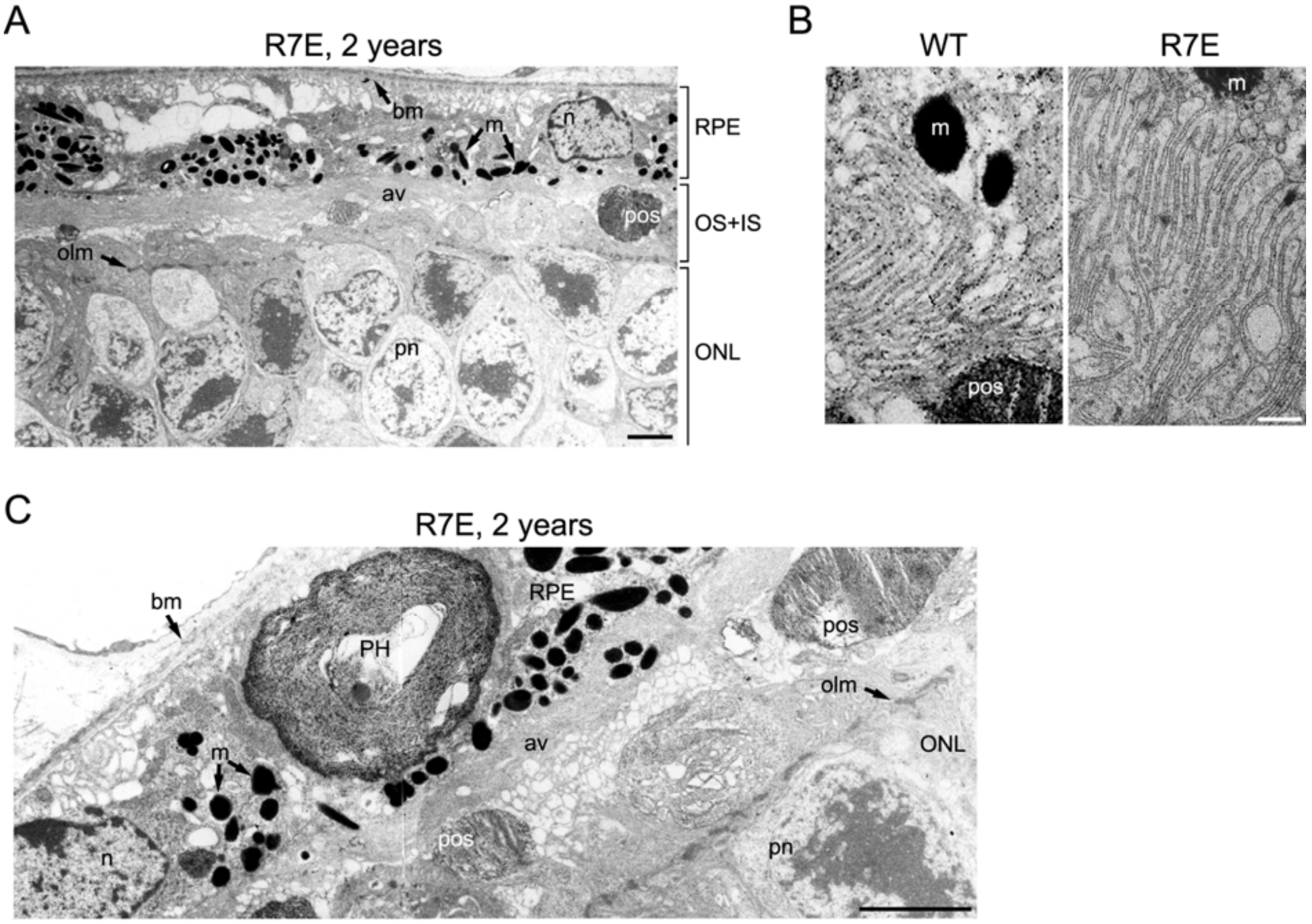


**Figure S2: Electron microscopy of 2-year-old R7E retina.**

A. Note the almost complete absence of outer and inner segments (OS + IS) as photoreceptor nuclei (ONL) are in close proximity to the retinal pigmented epithelium (RPE).

B-C. In both wild-type and R7E mice, cells from the retinal pigment epithelium displayed numerous apical microvilli contacting photoreceptor segments and showed typical outer segment phagocytosis (PH). These observations further indicate that rod outer segment loss in R7E mice is due to a defect in their renewal. Scale bars are 3 µm in A and B and 0.5 µm in C. Abbreviations: av, apical microvilli; bm, Bruch's basement membrane; IS, inner segments; m, melanin pigments; n, nuclei of RPE cells; olm, outer limiting membrane; OS, outer segments; ONL, outer nuclear layer; PH, phagosomes; pos, photoreceptor outer segments; pn, photoreceptor nuclei; RPE, retinal pigment epithelium.

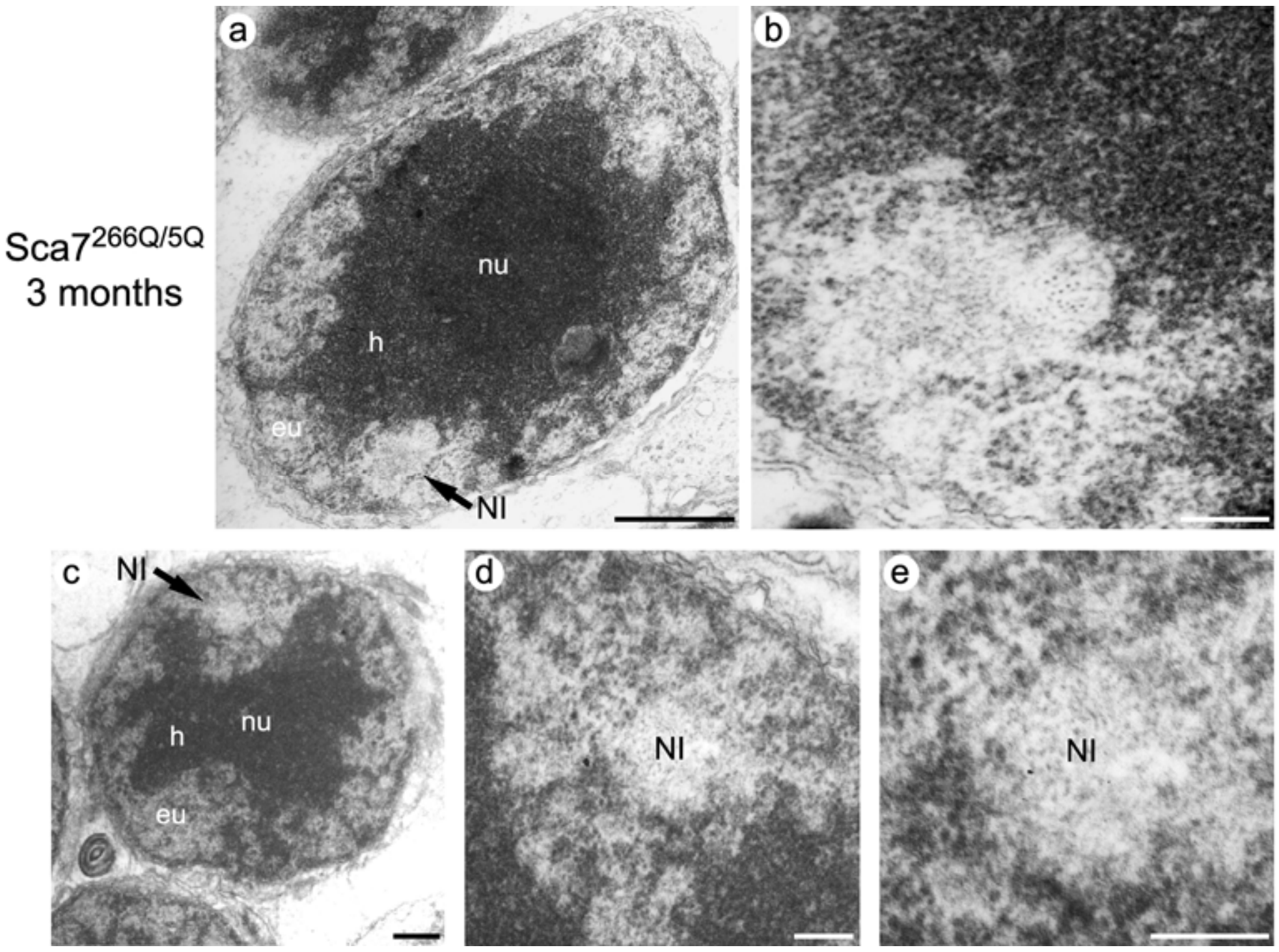


**Figure S3: Ultrastructure of NIs found in rod photoreceptors from 3-month-old Sca7$^{266Q/5Q}$ mice.**

Examples of nuclear inclusions (NI, indicated by large arrows in a and c) found within rods by conventional electron microscopy. At higher magnifications, the NI appeared as a pale stained structure surrounded by euchromatin (eu), distinct from the darkly stained adjacent heterochromatin (h) territory and central nucleolus (Nu). Scale bars are 1 µm in a, 0.25 µm in b, d, e and 0.5 µm in c. Abbreviations: eu, euchromatin; h, heterochromatin; NI, nuclear inclusion; nu, nucleolus.

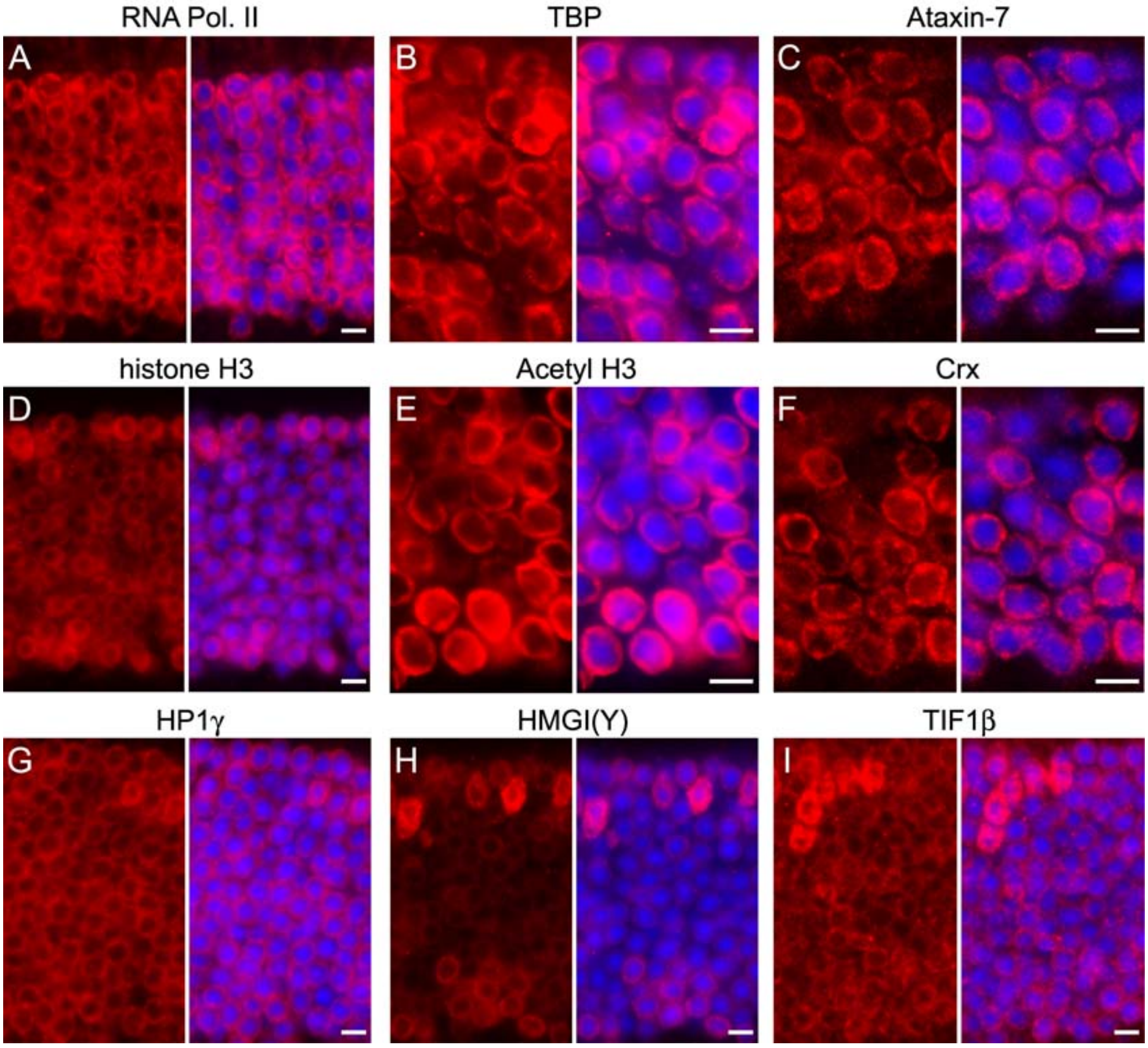


**Figure S4: Unique architecture of rod photoreceptor nuclei.**

Retinal cryosections (10 μm) from 2-month-old WT animals were stained using DAPI (blue) and antibodies against various nuclear proteins (red): RNA polymerase II (A), TBP (B), a TFTC/STAGA subunit ATXN7 (C), unmodified histone H3 (D), K9, K14 acetylated histone H3 (E), the photoreceptor specific transcriptional activator Crx (F), the heterochromatin protein HP1γ (G), the non-histone chromosomal protein HMGI(Y) (H) and a transcriptional repressor (TIF1β). Fluorescence images were collected and merged to visualize the intranuclear localization of several transcription factors and chromatin regulators in wild-type rod nuclei. All proteins analyzed present a characteristic ring-like appearance and are predominantly localized to the thin rim of peripheral euchromatin. Scale bar represents 5 μm.

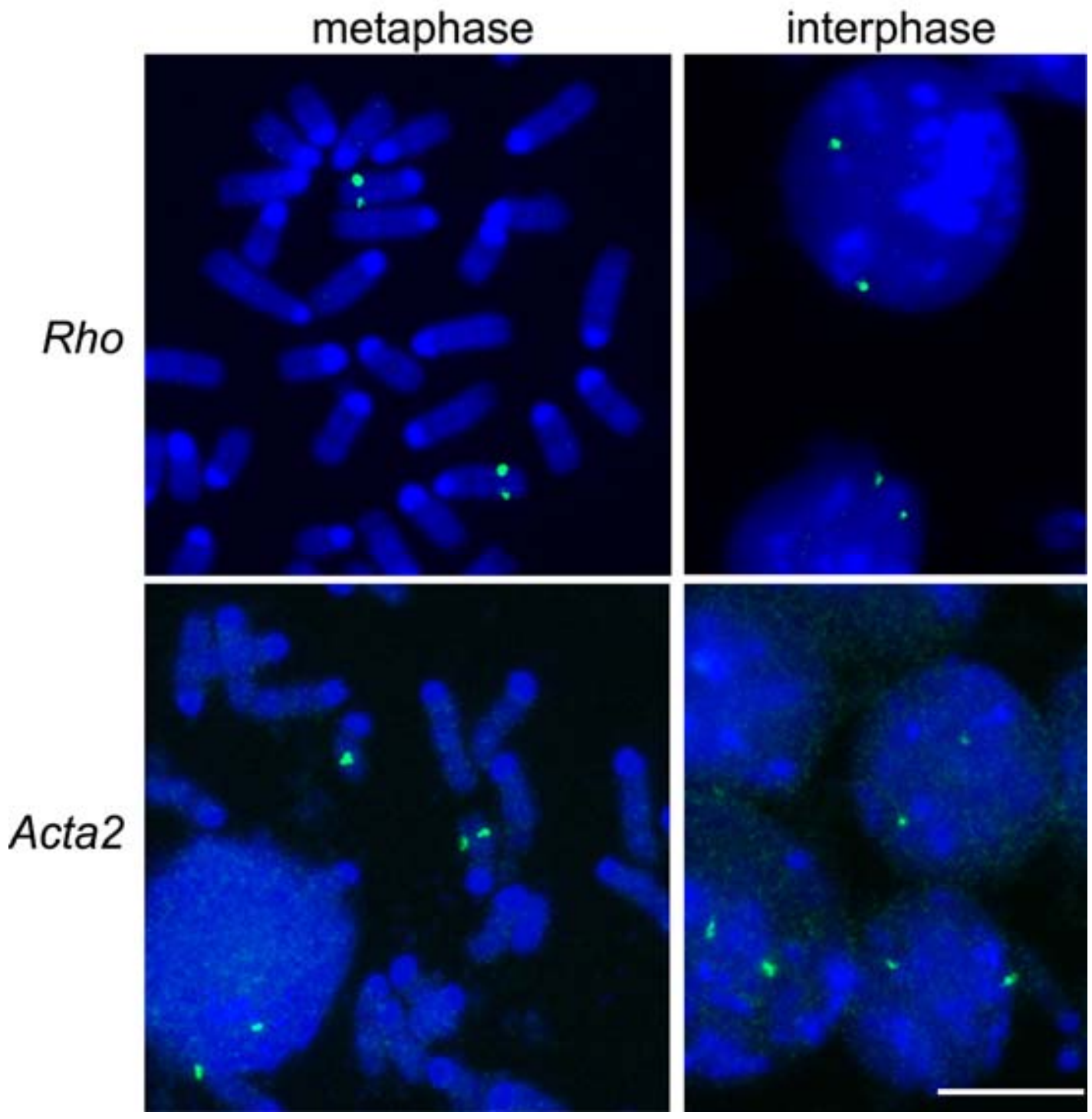


**Figure S5: Demonstration of FISH probes specificity.**

FISH of *Rho* and *Acta2* probes to mouse ES cell spreads revealed specific hybridization on metaphase chromosomes and interphase nuclei, counterstained with DAPI. Scale bar is 5 μm.

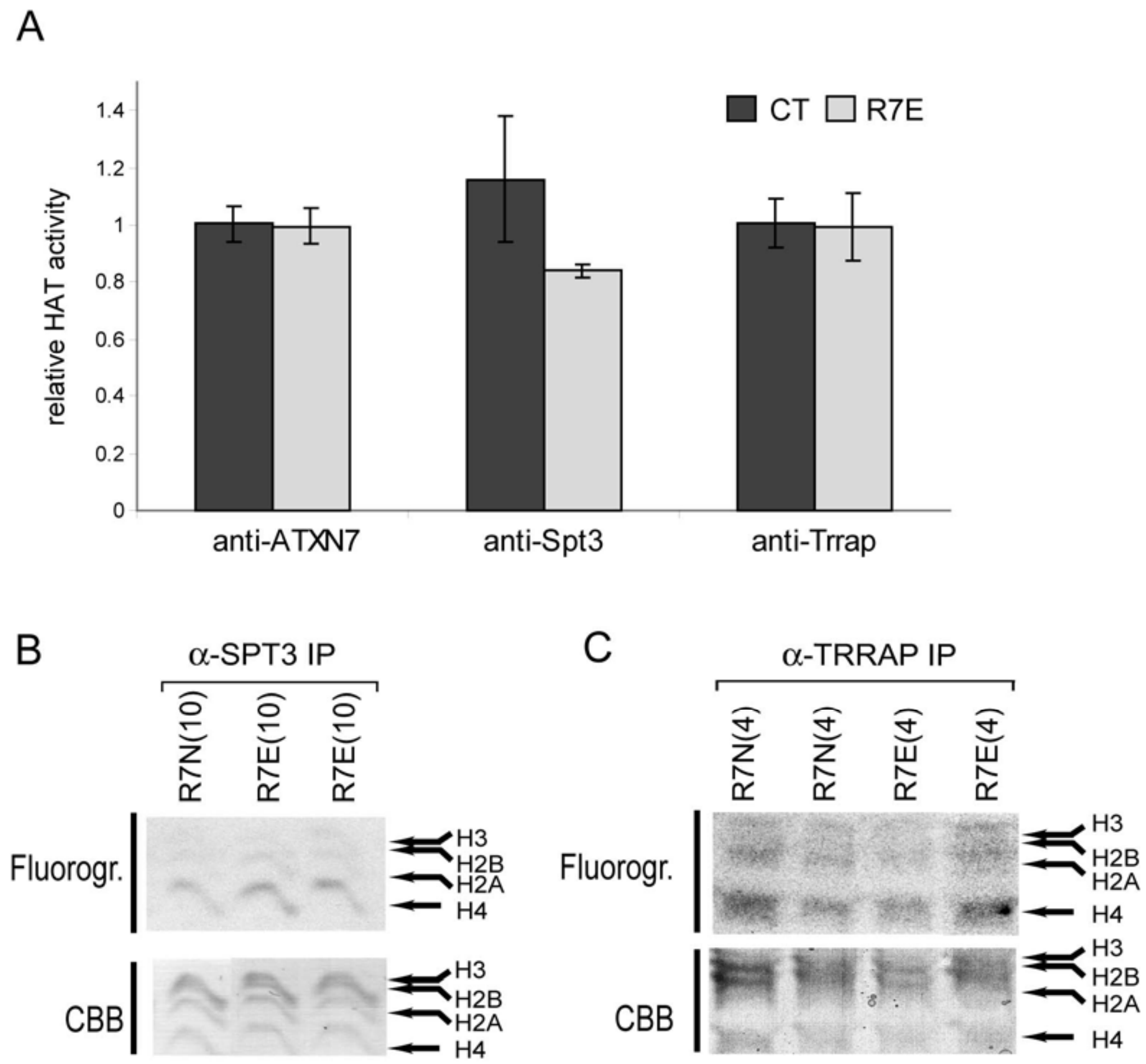


**Figure S6: TFTC histone acetyl transferase activity on histone or nucleosomal substrates is not altered by ATXN7 polyglutamine expansion in R7E retina.**

A. Quantification of the HAT activities of TFTC-type complexes immunopurified from R7N and R7E retina. HAT assays were performed after immunopurifications using anti-ATXN7, anti-SPT3 or anti-TRRAP antibodies, as indicated in Figure 6. No significant differences could be detected when comparing R7N and R7E mice. Each HAT activity was calculated and is represented as a ratio to the mean of all animals analyzed in each assay. Each bar represent the mean value ± SEM (*n* = 2-7).

B-C. HAT activities of immunopurified complexes bound to the anti-SPT3 mAb (B) or anti-TRRAP mAb (C) beads from R7N and R7E retina were measured by an *in vitro* HAT assay performed on oligonucleosomes prepared from HeLa cells. Histones were separated by SDS-

PAGE and acetylated histones were visualized by fluorography (Fluorgr.). The position of each histone is indicated. The age of the mice are indicated in weeks in brackets.

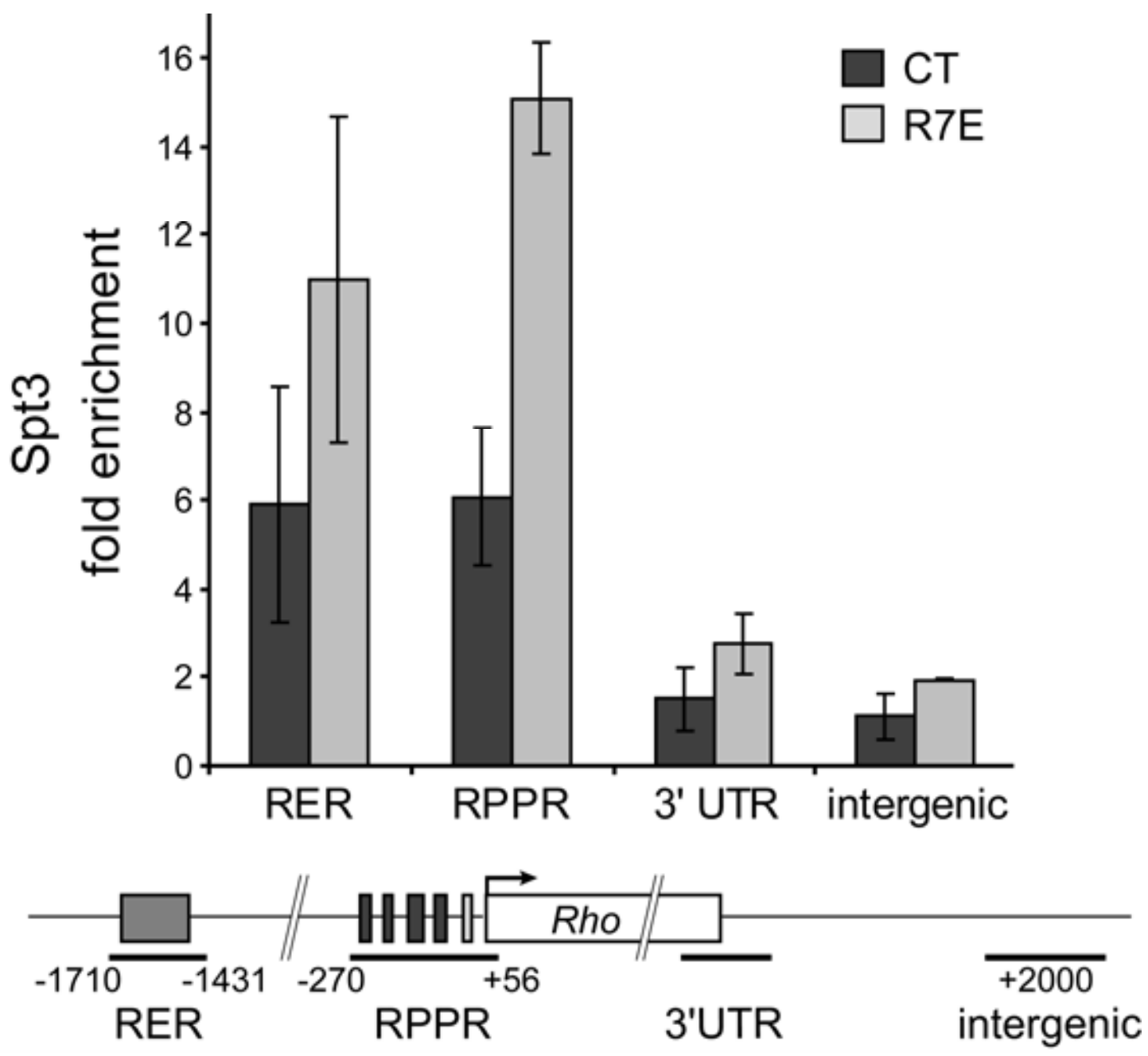


**Figure S7: Increased recruitment of TFTC-type complexes containing polyglutamine expanded ATXN7 at different regions of the *Rho* gene.**

Chromatin immunoprecipitation assays (ChIP) were performed using formaldehyde-fixed chromatin extracts of retina from 2 month-old control (WT or R7N; dark grey) and R7E (light grey) mice. Specific regions within the *Rho* gene were analyzed by real-time PCR, after ChIP using an antibody against a TFTC-specific subunit (Spt3). Quantification of fold enrichment was calculated as indicated in Figure S9. Each bar represents the mean value ± SEM (*n* = 4-6).

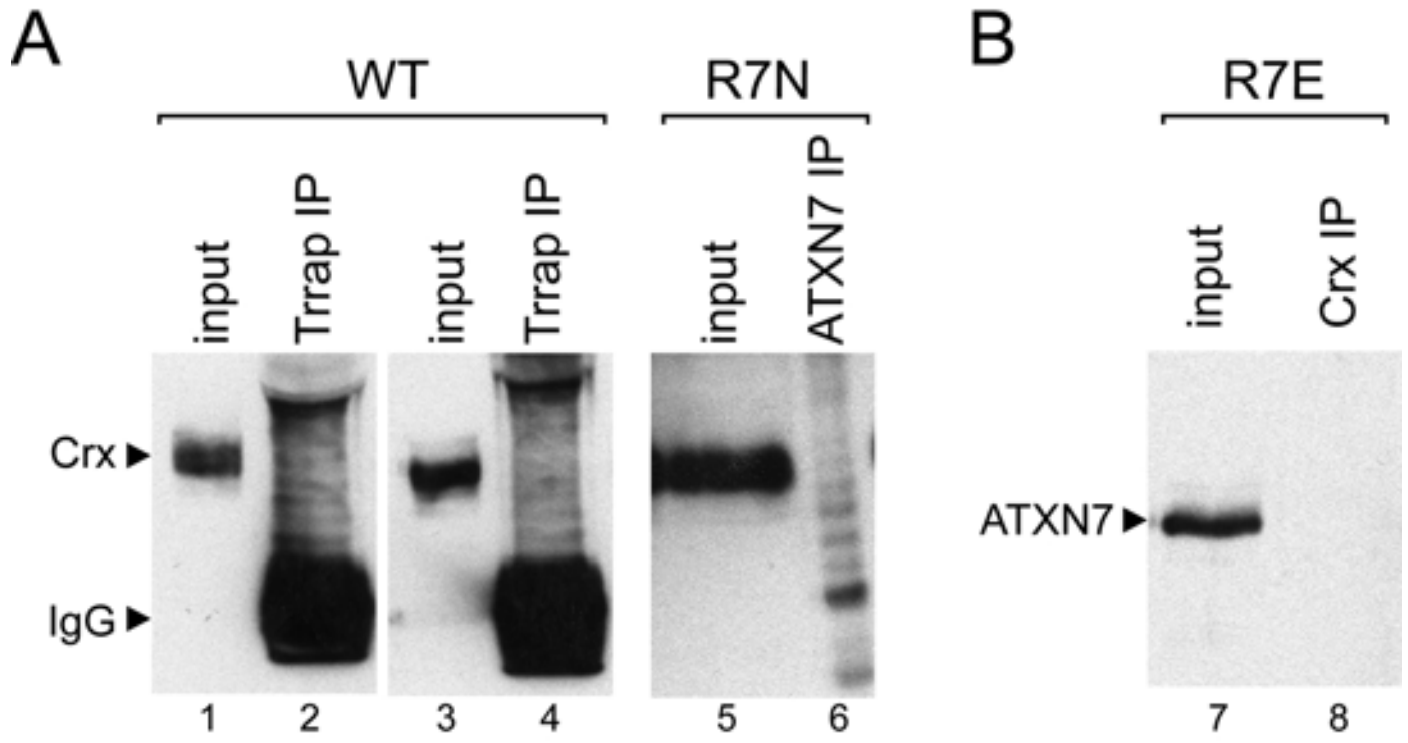


**Figure S8: Absence of *in vivo* interaction of Crx with TFTC/STAGA complexes.**

(A) Whole retinal extracts from WT or normal ATXN7 expressing mice (R7N) were immunoprecipitated with two distinct anti-Trrap polyclonal antibodies (lanes 2 and 4) or one anti-ATXN7 monoclonal antibody (lane 6). Immunoprecipitated complexes retained on protein A–sepharose beads were analyzed by immunoblotting with a polyclonal antibody detecting Crx. Similar experiments were performed using the anti-Crx polyclonal antibody for immunoprecipitation and a monoclonal anti-ATXN7 antibody for immunoblot analysis. Both panels demonstrated absence of co-immunprecipitaion of these Crx with either Trrap or ATXN7 in mouse retina (lanes 2, 4, 6 and 8). IgG bands were detected with the secondary anti-rabbit peroxidase-conjugated antibodies (lanes 2 and 4). Input (lanes 1, 3, 5 and 7) represents 10% of the amount of whole retinal extracts used for immunoprecipitation.

A

$$\text{fold enrichment of protein X} = \frac{\left([DNA]_{IP} / [DNA]_{input}\right)_{Ab\ X}}{\left([DNA]_{IP} / [DNA]_{input}\right)_{no\ Ab}}$$

B

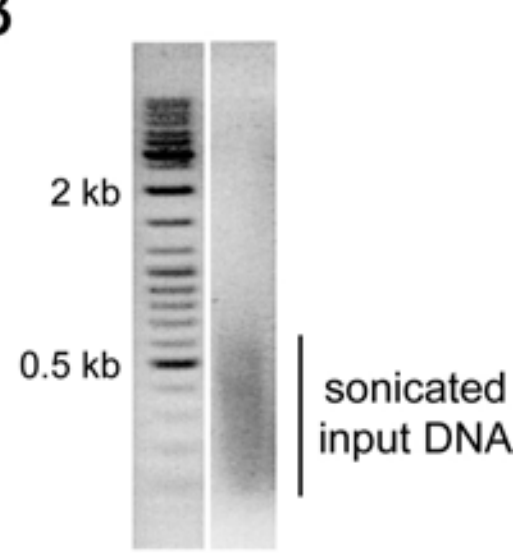


**Figure S9: Chromatin immunoprecipitation experiment design.**

A. Quantification of the fold enrichment for each antibody at specific loci was obtained by calculating the ratio of immunoprecipitated DNA relative to input DNA amounts, normalized to the ratio obtained with a control IP (no antibody).

B. Representative example of sonicated input chromatin isolated from mouse retina (see Materials and Methods).
