# Supplementary material for: Glutamine-Expanded Ataxin-7 Alters TFTC/STAGA Recruitment and Chromatin Structure Leading to Photoreceptor Dysfunction
Source: PLoS Biol. 2006 Feb 28;4(3):e67. doi: 10.1371/journal.pbio.0040067 (PMC1382020; doi:10.1371/journal.pbio.0040067)
Supplement: Supplementary file 9 [file pbio.0040067.sg009.pdf]

A

$$\text{fold enrichment of protein X} = \frac{\left([DNA]_{IP} / [DNA]_{input}\right)_{Ab\ X}}{\left([DNA]_{IP} / [DNA]_{input}\right)_{no\ Ab}}$$

B

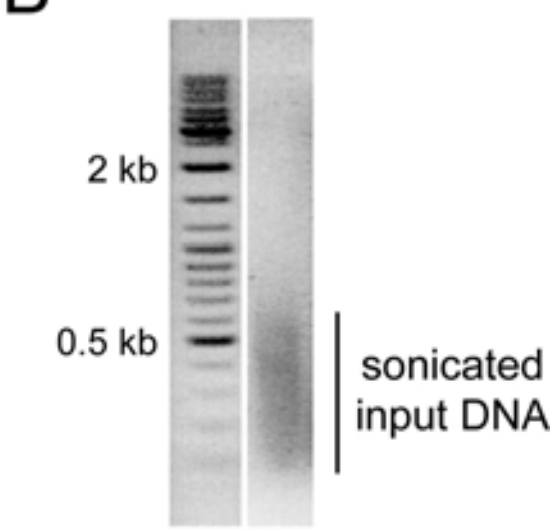

Figure S9: Chromatin immunoprecipitation experiment design.

A. Quantification of the fold enrichment for each antibody at specific loci was obtained by calculating the ratio of immunoprecipitated DNA relative to input DNA amounts, normalized to the ratio obtained with a control IP (no antibody).

B. Representative example of sonicated input chromatin isolated from mouse retina (see Materials and Methods).
