# Supplementary material for: Glutamine-Expanded Ataxin-7 Alters TFTC/STAGA Recruitment and Chromatin Structure Leading to Photoreceptor Dysfunction
Source: PLoS Biol. 2006 Feb 28;4(3):e67. doi: 10.1371/journal.pbio.0040067 (PMC1382020; doi:10.1371/journal.pbio.0040067)
Supplement: Supplementary file 10 [file pbio.0040067.st001.doc]

**Table S1: Comprehensive list of deregulated genes in retina from 2 month-old R7E mice.**

| **Photoreceptor-specific genes** | | **Refseq accession numbers** | **Relative**  **fold change***^a^* | **Function** |
| --- | --- | --- | --- | --- |
| Rhodopsin | Rho | NM_145383 | down 10x | Rod  phototransduction   |
| Transducin-α | Gnat1 | NM_008140 | down 10x |  |
| cGMP phosphodiesterase | Pde6b | NM_008806 | down 10x |  |
| Phosducin | Pdc | NM_024458 | down 10x |  |
| cGMP-gated channel | Cncg | NM_007723 | down 5x |  |
| Arrestin | Sag | NM_009118 | down 5x |  |
| Rhodopsin kinase | Rhok | NM_011881 | down 5x |  |
| Guanylate cylase activator | Guca | NM_008189 | down 2x |  |
| Recoverin | Rcvrn | NM_009038 | down 5x |  |
| Retinal-binding protein | Rbp3 | NM_015745 | down 2x |  |
| Rod outer segment membrane protein 1 | Rom1 | NM_009073 | down 5x | Rod  structural components |
| Peripherin 2 | Prph2 | NM_008938 | down 5x |  |
| Neural retina leucine zipper | Nrl | NM_008736 | down 3x | Transcription factors |
| Nuclear receptor Nr2e3 | Nr2e3 | NM_013708 | down 5x |  |
| Cone-rod homeobox | Crx | NM_007770 | down 2x |  |
| Tubby-like protein 1 | Tulp1 | [NM_021478](http://www.ncbi.nlm.nih.gov/entrez/viewer.fcgi?db=Nucleotide&dopt=GenBank&val=33468972) | no difference |  |
| Blue cone opsin | Bcp | NM_007538 | down 2x | Cone  phototransduction |
| Cone transducin | Gnat2 | NM_008141 | up 2x |  |
| Cone arrestin | Carr | NM_133205 | down 1.5x |  |

*^a^*Results were compiled from Northern blot and/or real-time RT-PCR experiments performed on at least three independent control (WT or R7N) and R7E samples.

**Supplemental References**

1. Yvert G, Lindenberg KS, Picaud S, Landwehrmeyer GB, Sahel JA, et al. (2000) Expanded polyglutamines induce neurodegeneration and trans-neuronal alterations in cerebellum and retina of SCA7 transgenic mice. Hum Mol Genet 9: 2491-2506.

2. Helmlinger D, Hardy S, Sasorith S, Klein F, Robert F, et al. (2004) Ataxin-7 is a subunit of GCN5 histone acetyltransferase-containing complexes. Hum Mol Genet 13: 1257-1265.

3. Brand M, Moggs JG, Oulad-Abdelghani M, Lejeune F, Dilworth FJ, et al. (2001) UV-damaged DNA-binding protein in the TFTC complex links DNA damage recognition to nucleosome acetylation. Embo J 20: 3187-3196.

4. Acker J, de Graaff M, Cheynel I, Khazak V, Kedinger C, et al. (1997) Interactions between the human RNA polymerase II subunits. J Biol Chem 272: 16815-16821.

5. Wieczorek E, Brand M, Jacq X, Tora L (1998) Function of TAF(II)-containing complex without TBP in transcription by RNA polymerase II. Nature 393: 187-191.

6. Chau KY, Munshi N, Keane-Myers A, Cheung-Chau KW, Tai AK, et al. (2000) The architectural transcription factor high mobility group I(Y) participates in photoreceptor-specific gene expression. J Neurosci 20: 7317-7324.

7. Nielsen AL, Ortiz JA, You J, Oulad-Abdelghani M, Khechumian R, et al. (1999) Interaction with members of the heterochromatin protein 1 (HP1) family and histone deacetylation are differentially involved in transcriptional silencing by members of the TIF1 family. Embo J 18: 6385-6395.

8. Zhu X, Craft CM (2000) Modulation of CRX transactivation activity by phosducin isoforms. Mol Cell Biol 20: 5216-5226.

9. Hicks D, Molday RS (1986) Differential immunogold-dextran labeling of bovine and frog rod and cone cells using monoclonal antibodies against bovine rhodopsin. Exp Eye Res 42: 55-71.
