# Supplementary material for: Glutamine-Expanded Ataxin-7 Alters TFTC/STAGA Recruitment and Chromatin Structure Leading to Photoreceptor Dysfunction
Source: PLoS Biol. 2006 Feb 28;4(3):e67. doi: 10.1371/journal.pbio.0040067 (PMC1382020; doi:10.1371/journal.pbio.0040067)
Supplement: Supplementary file 11 [file pbio.0040067.st002.doc]

**Table S2: Source of antibodies used and working dilutions.**

| **Antibody** | **Dilution** | **For***^a^* | **Source** |
| --- | --- | --- | --- |
| ATXN7 (1261) | 1:100 | IHF | [1] |
| ATXN7 (1598) | 1:100 | IP | [1] |
| ATXN7 (3SCA-1C1) | 1:1000 | WB | [1] |
| TRRAP (1930) | 1:100 | IP | [2] |
| TRRAP (2TRR-2D5) | 1:1000 | WB | [2] |
| GCN5 (2GC-2C11) | 1:1000 | WB | [3] |
| SPT3 (1SP-2C10) | 1:100-1:200 | IP, ChIP | [3] |
| RNA Pol II CTD (1PB-7G5) | 1:250 | ChIP | [4] |
| Acetyl K9 K14  histone H3 (06-599) | 1:100 | ChIP | Upstate |
| Histone H3 (06-755) | 1:100 | IHF | Upstate |
| TBP (3TF1-3G3) | 1:500 | IHF | [5] |
| HMGI(Y) | 1:100 | IHF | [6] |
| HP1γ (2 Mod-1G6) | 1:500 | IHF | [7] |
| TIF1β (1Tb3) | 1:500 | IHF | [7] |
| Crx | 1:100 | WB, IP, IHF | [8] |
| Rhodopsin (4D2) | 1:10,000 | IHF, IHC | [9] |

*^a^*Abbreviations used are: WB, western blot; IP, immunoprecipitation; ChIP, chromatin immunoprecipitation; IHF, immunohistofluorescence; IHC, immunohistochemistry.

**Supplemental References**

1. Yvert G, Lindenberg KS, Picaud S, Landwehrmeyer GB, Sahel JA, et al. (2000) Expanded polyglutamines induce neurodegeneration and trans-neuronal alterations in cerebellum and retina of SCA7 transgenic mice. Hum Mol Genet 9: 2491-2506.

2. Helmlinger D, Hardy S, Sasorith S, Klein F, Robert F, et al. (2004) Ataxin-7 is a subunit of GCN5 histone acetyltransferase-containing complexes. Hum Mol Genet 13: 1257-1265.

3. Brand M, Moggs JG, Oulad-Abdelghani M, Lejeune F, Dilworth FJ, et al. (2001) UV-damaged DNA-binding protein in the TFTC complex links DNA damage recognition to nucleosome acetylation. Embo J 20: 3187-3196.

4. Acker J, de Graaff M, Cheynel I, Khazak V, Kedinger C, et al. (1997) Interactions between the human RNA polymerase II subunits. J Biol Chem 272: 16815-16821.

5. Wieczorek E, Brand M, Jacq X, Tora L (1998) Function of TAF(II)-containing complex without TBP in transcription by RNA polymerase II. Nature 393: 187-191.

6. Chau KY, Munshi N, Keane-Myers A, Cheung-Chau KW, Tai AK, et al. (2000) The architectural transcription factor high mobility group I(Y) participates in photoreceptor-specific gene expression. J Neurosci 20: 7317-7324.

7. Nielsen AL, Ortiz JA, You J, Oulad-Abdelghani M, Khechumian R, et al. (1999) Interaction with members of the heterochromatin protein 1 (HP1) family and histone deacetylation are differentially involved in transcriptional silencing by members of the TIF1 family. Embo J 18: 6385-6395.

8. Zhu X, Craft CM (2000) Modulation of CRX transactivation activity by phosducin isoforms. Mol Cell Biol 20: 5216-5226.

9. Hicks D, Molday RS (1986) Differential immunogold-dextran labeling of bovine and frog rod and cone cells using monoclonal antibodies against bovine rhodopsin. Exp Eye Res 42: 55-71.
